# Genome-wide identification and expression analysis of jasmonate ZIM domain gene family in tuber mustard (*Brassica juncea* var. *tumida*)

**DOI:** 10.1371/journal.pone.0234738

**Published:** 2020-06-16

**Authors:** Zhaoming Cai, Yuanqing Chen, Jingjing Liao, Diandong Wang

**Affiliations:** College of Life Science and Technology, Yangtze Normal University, Chongqing, P.R. China; Huazhong University of Science and Technology, CHINA

## Abstract

Tuber mustard, which is the raw material of Fuling pickle, is a crop with great economic value. However, during growth and development, tuber mustard is frequently attacked by the pathogen *Plasmodiophora brassicae* and frequently experiences salinity stress. Jasmonic acid (JA) is a hormone related to plant resistance to biotic and abiotic stress. Jasmonate ZIM domain proteins (JAZs) are crucial components of the JA signaling pathway and play important roles in plant responses to biotic and abiotic stress. To date, no information is available about the characteristics of the *JAZ* family genes in tuber mustard. Here, 38 *BjJAZ* genes were identified in the whole genome of tuber mustard. The *BjJAZ* genes are located on 17 of 18 chromosomes in the tuber mustard genome. The gene structures and protein motifs of the *BjJAZ* genes are conserved between tuber mustard and *Arabidopsis*. The results of qRT-PCR analysis showed that *BjuA030800* was specifically expressed in root, and *BjuA007483* was specifically expressed in leaf. In addition, 13 *BjJAZ* genes were transiently induced by *P*. *brassicae* at 12 h, and 7 *BjJAZ* genes were induced by salt stress from 12 to 24 h. These results provide valuable information for further studies on the role of *BjJAZ* genes in the regulation of plant growth and development and in the response to biotic and abiotic stress.

## Introduction

Tuber mustard is the raw material of Fuling pickle, which has great economic value. As a Cruciferae plant species, tuber mustard is frequently attacked by soil pathogens, such as *Plasmodiophora brassicae*. Discovering the mechanism by which tuber mustard responds to pathogens is important for improving the production of this crop. Jasmonic acid (JA) is a plant hormone that is responsible for plant resistance to biotic and abiotic stress. Studying the roles of JA signaling-related genes in the regulation of plant growth and development will shed light on the molecular mechanism underlying the resistance of tuber mustard to pathogens such as *P*. *brassicae*.

JA is an endogenous growth-regulating substance found in higher plants. Methyl ester JA (MeJA) and isoleucine-conjugated JA (JA-Ile) are derivatives of JA and have similar functions to JA. Collectively, these hormones are known as jasmonates. Jasmonates are involved in many biological processes, such as plant growth and development [1–3] and plant responses to herbivore damage [4]. JAs also play roles in plant responses to biotic stress, such as the response of *Brassica*. *napus* to *P*. *brassicae* [5], and plant responses to abiotic stress, such as the salinity tolerance of wheat [6]. JA functions through a cascade of molecular signal transductions, and the Coronatine Insensitive1(COI1) and jasmonate ZIM domain (JAZ) proteins are the coreceptors of JA-Ile [7]. The interaction between COI1 and JAZ proteins activated by JA-Ile leads to the degradation of JAZ proteins by the 26S proteasome, which eventually activates the function of some JA-responsive transcription factors (TFs), such as MYCs, MYBs, NACs, ERFs and WRKYs [8–9], resulting in JA-related responses.

JAZ proteins, which contain a ZIM domain (TIFY) at the N-terminus, are repressors of the JA signaling pathway and exhibit homo- and heteromeric interactions [10]. JAZ proteins play roles in plant responses to biotic and abiotic stress. It has been reported that overexpression of *TaJAZ1* led to enhanced resistance against powdery mildew in bread wheat [11]. In rice, *OsJAZ8* was reported to be involved in the induction of monoterpene linalool, which conferred resistance to rice bacterial blight [12]. Some *PtJAZ* genes, including *PtJAZ3*, *PtJAZ6*, *PtJAZ9* and *PtJAZ15*, were found to be induced by leaf rust pathogen infection in poplar, suggesting their potential roles in plant responses to abiotic stresses [13]. In *Arabidopsis*, *jaz10* mutants exhibit enhanced susceptibility to the pathogen *Pseudomonas syringae DC3000* [14]. These studies suggest that JAZ proteins play crucial roles in plant resistance to biotic stress. In addition, JAZ proteins are widely involved in plant tolerance to salt stress. Overexpression of *GhJAZ2* resulted in enhanced sensitivity to salt stress in transgenic cotton [15]. Overexpression of *OsJAZ8* in rice led to improved salt tolerance in transgenic seedlings [16].

JAZ proteins also exist in tuber mustard; however, the functions of *JAZ* family genes in this crop are still unknown. Here, we identified *JAZ* family members in tuber mustard and analyzed their gene and protein characteristics. We also investigated the expression patterns of the *BjJAZ* genes during plant growth and development and their expression profiles in the roots of tuber mustard seedlings under pathogen and salinity treatments. This study will be useful for functional studies of JAZs in tuber mustard.

## Materials and methods

### Plant materials and growth conditions

To evaluate the gene expression patterns in different tissues, seedlings of the tuber mustard cultivar ‘Yong An XiaoYe’ were planted in a field for six months and grown to the reproductive stage, and the roots, stems, leaves, flowers and pods were sampled for qRT-PCR analysis. For stress treatments, seeds of the tuber mustard cultivar ‘Yong An XiaoYe’ were sown in plant pots in vermiculite and turfy soil (2:1) medium, and the culture temperature was 22°C with a 16/8 h light/dark regime. For the salt-stress treatments, two-week-old seedlings were irrigated with a nutrient solution with or without 200 mM NaCl for 0, 6, 12, 24 and 48 h, and the roots were sampled for qRT-PCR analysis. For pathogen treatment, two-week-old seedlings were inoculated with a 5-mL resting spore suspension of *P*. *brassicae* (OD_600_ = 0.07) for 0, 12, 24, 36 and 72 h, and the roots were sampled for qRT-PCR analysis.

### Identification of BjJAZ proteins in tuber mustard and bioinformatic analysis

The JAZ protein sequences of *A*. *thaliana* were obtained from the *Arabidopsis* information resource website (https://www.arabidopsis.org/), the gene ID and protein sequences of these *AtJAZ* genes are listed in S1 File. A BLASTP analysis was performed using the AtJAZ proteins as the query sequences to search the putative JAZ proteins in the *Brassica*. *juncea* genome database (chromosome V1.5, http://brassicadb.org/brad/), the parameters were set as Evalue < 1e-10, identity > 70%, query coverage > 95%, and the other parameters were defaulted. The Pfam database [17] (Pfam; http://pfam.xfam.org/)was used to analyze the conserved domains of all the selected nonredundant sequences, and the proteins that contained both the TIFY domain and Jas domain were defined as belonging to the JAZ family. For gene location analysis, the region of each *BjJAZ* genomic sequence was downloaded from the *B*. *juncea* genome database, and the location of each *BjJAZ* gene was marked in the tuber mustard chromosome using MapDraw V2.1 software [18]. The coding sequence and corresponding genomic sequences of *BjJAZ* were downloaded from the *B*. *juncea* genome database, and the exon-intron structure diagram was drawn using online software for the GSDS2.0 server (http://gsds.cbi.pku.edu.cn/). The phylogenetic analysis was performed using MEGA5 software with the neighbor-joining method [19], the bootstrap value was used for 1000 replicates, and the “p-distance model” and “Pairwise deletion” were used for “Model/Method” and “Gaps/Missing” data treatment, respectively. The amino acid isoelectric point (pI) and molecular weight (MW) were predicted with ExPASy software (https://web.expasy.org/compute_pi/), and protein motifs were analyzed with the NCBI-CDD (National Center for Biotechnology Information, https://www.ncbi.nlm.nih.gov/Structure/cdd/wrpsb.cgi). The diagram of the protein motifs was drawn by ExPASy (https://prosite.expasy.org/prosite.html), and the sequence logo for the TIFY domain and Jas domain was generated with WebLogo 3 (http://weblogo.berkeley.edu/). The comparison and annotation of orthologous gene clusters of the *JAZ* genes among tuber mustard and *Arabidopsis* were analyzed using the online software OrthoVenn2 under default parameters [20]. The duplication events were analyzed by performing BLASTP for proteins against each other, which were defined when both the identity and query coverage was > 80% between partner sequences [21,22]. Tandem duplication genes were defined according to the methods of Holub, where a chromosomal region within 200 kb containing two or more genes is defined as a tandem duplication event [23]. The online software MEME (Version 5.1.1; http://meme-suite.org/tools/meme) was used to elucidate conserved motifs of BjJAZs, the maximum number of motifs was 9, and the other parameters were defaulted [24].

### RNA extraction and qRT-PCR analysis

Total RNA was extracted from different plant materials using TRIzol reagent (Tiangen Biotech (Beijing) Co., Ltd., China) following the manufacturer’s instructions, and the concentration and quality was detected using Nanodrop One (Thermo Fisher Scientific, USA) and 1% agarose gel electrophoresis. The total RNA samples were treated with DNase I (Invitrogen Biotech Co., Ltd., USA) to remove contaminating genomic DNA. First-strand cDNA was synthesized from 500 ng RNA in 20 μL volume using a FastQuant RT Kit (Tiangen Biotech (Beijing) Co., Ltd., China) following the manufacturer’s instructions. qRT-PCR was performed using SuperReal PreMix Plus (SYBR Green; Tiangen Biotech (Beijing) Co., Ltd., China). For qRT-PCR analysis, each reaction was performed in 10 μL volume containing 0.3 μL diluted (50x) cDNA sample as template, 5 μL SuperReal PreMix Plus, 0.5 μL (10 μmol/L) each of forward and reverse primers, and 3.7 μL ddH_2_O. The prepared samples were subjected to the following conditions: 95°C for 15 min, followed by 40 cycles at 95°C for 10 s, 60°C for 20 s, and 72°C for 30 s. The fluorescence was determined observing the last step of each cycle, and the Melting Curves program was added in the last step. LightCycler 480 II (Roche, Germany) was used for amplification detection. Each of three biological and technical replicates was examined for each stress treatment. The 18S rRNA gene (*BjuA046942*) was used as the internal reference gene for qRT-PCR, and the gene-specific primers are listed in S1 Table.

### Statistical analysis

The qRT-PCR gene relative expression levels were calculated using the 2^−ΔΔCt^ method and the data were analyzed using SigmaPlot 10.0 (Systat Software Inc., USA) and SPSS 16.0 software (IBM Corporation, USA). The averages and standard deviations of all results were calculated using SPSS 16.0 software, and one-way ANOVA followed by the LSD test was used for difference significance analysis. Significant differences are designated as follows: *, *p* < 0.05; **, *p* < 0.01; and ***, *p* < 0.001.

## Results

### Identification of *JAZ* family genes in tuber mustard

To identify the *BjJAZ* genes in the tuber mustard genome, protein sequence BLAST searches were performed in the Brassica Database using the *Arabidopsis* JAZ protein sequences as the queries. The protein sequences in the tuber mustard genome with more than 70% identity to AtJAZ proteins were selected as the candidate homologs of JAZs, and the JAZ proteins were further identified by gene structure and protein domain conservation analyses. A total of 38 nonredundant *BjJAZ* genes were identified in the genome of tuber mustard, and the identification was supported by conserved functional domain and multiple sequence alignment analyses. The sequences of the predicted BjJAZ proteins and gene IDs are listed in S2 File. The gene and protein characteristics of the identified *JAZ* genes were investigated by bioinformatic analysis. The lengths of the *BjJAZ* genomic sequences ranged from 458 bp to 2929 bp, and the lengths of the protein-coding sequences (CDSs) ranged from 369 bp to 1065 bp. The predicted lengths of the proteins encoded by the *BjJAZ* genes varied from 122 amino acids to 354 amino acids, with the corresponding molecular weights (MW) ranging from 13.74 kDa to 37.50 kDa, and the theoretical isoelectric (pI) values ranged from 5.20 to 10.33 (Table 1).

**Table 1:** The *BjJAZ* family genes in tuber mustard.

Sequence ID	Locus	Genomics (bp)	CDS (bp)	Protein (aa)	pI	MW (kD)
BjuB032431	B03	1403	822	273	9.49	29.82
BjuB031487	B03	1411	825	274	9.49	30.09
BjuA025820	A06	1296	762	253	9.73	27.15
BjuB043409	B03	1047	756	251	9.22	27.33
BjuA030800	A08	1121	780	259	9.49	28.31
BjuB021388	B06	1185	687	228	9.01	25.11
BjuA027037	A07	1170	675	224	9.18	24.49
BjuA029428	A07	1150	738	245	9.20	26.63
BjuB029798	B03	1213	759	252	9.36	27.68
BjuA005572	A02	1199	723	240	9.23	26.07
BjuB011370	B05	1246	738	245	9.01	26.72
BjuA045157	A01	2929	1062	353	9.49	37.44
BjuB007213	B07	2676	1065	354	9.53	37.50
BjuA046021	A05	2574	1008	335	9.51	35.87
BjuB025543	B01	2602	1020	339	9.47	36.17
BjuB043343	B03	1269	786	261	9.14	29.18
BjuA022138	A06	1287	771	256	9.28	28.75
BjuB029203	B04	1231	771	256	10.33	28.96
BjuA030507	A08	1286	813	270	8.32	30.03
BjuA001107	A09	1137	798	265	9.71	29.88
BjuB030035	B03	1886	684	227	9.21	25.45
BjuB010656	B05	1338	819	272	8.99	30.33
BjuA007483	A02	1320	822	273	8.95	30.18
BjuB026559	B01	458	369	122	9.27	13.74
BjuB032915	B03	586	396	131	9.69	15.04
BjuB029529	B04	568	396	131	9.23	15.07
BjuA034780	A09	579	402	133	9.47	15.39
BjuA007387	A02	2078	822	273	9.82	29.27
BjuA027135	A07	1133	639	212	9.17	23.50
BjuB030369	B03	1935	834	277	9.85	29.88
BjuA027422	A07	2148	804	267	9.76	28.81
BjuB035964	B02	1605	591	196	9.90	21.87
BjuA041687	A03	1486	588	195	9.95	21.79
BjuB014771	B08	1673	588	195	9.80	21.79
BjuA022588	A06	1341	666	221	5.20	23.10
BjuO008948	Contig	1108	633	210	8.93	22.45
BjuA047148	A10	1079	612	203	6.92	21.43
BjuA001950	A05	549	372	123	9.90	14.01

### Phylogenetic analysis of BjJAZs

**Fig 1:**
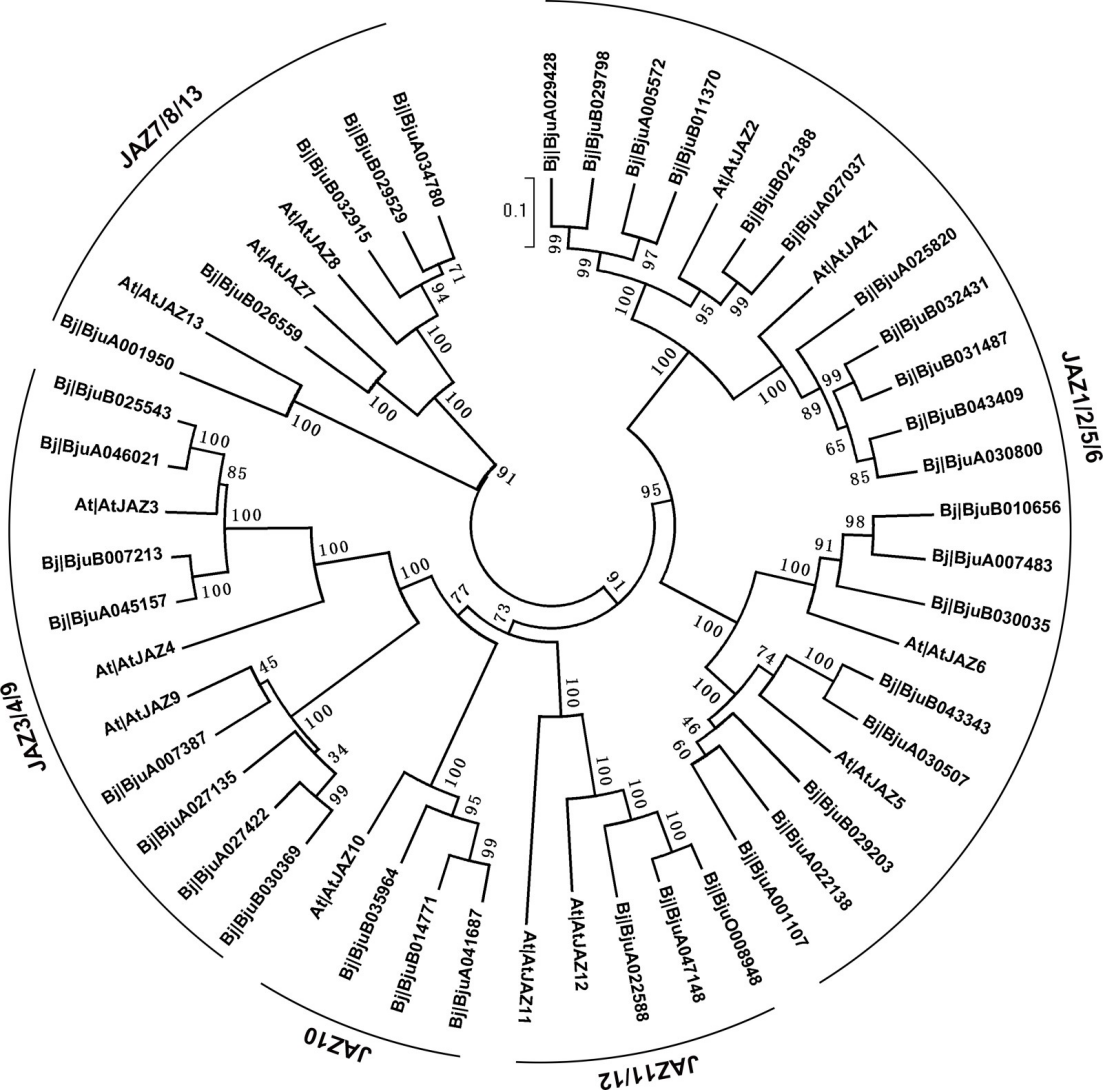
Phylogenetic analysis of *JAZ*s in *Arabidopsis thaliana* and tuber mustard. The protein sequence of each gene was used for the alignment, and the phylogenetic neighbor-joining tree was constructed using MEGA5 phylogenetic analysis software.

To characterize the evolutionary relationships among the *JAZ* genes in tuber mustard and *Arabidopsis*, a phylogenetic analysis was performed using MEGA 5 software, and the BjJAZ and AtJAZ protein sequences were used for the analysis. As shown in the neighbor-joining phylogenetic tree, these JAZs were assigned tofive separate clades: JAZ1/2/5/6, JAZ11/12, JAZ7/8/13, JAZ10, and JAZ3/4/9 (Fig 1), and the details are listed in Table 1. We also analyzed the orthologous relationships among the *BjJAZ* and *AtJAZ* genes using the online software OrthoVenn2, and 13 clusters were identified, of those, 11 orthologous clusters were found shared between tuber mustard and *Arabidopsis*, expect for 2 single-copy gene clusters (S3 File).

### Chromosomal locations of *BjJAZs* in the genome of tuber mustard

**Fig 2:**
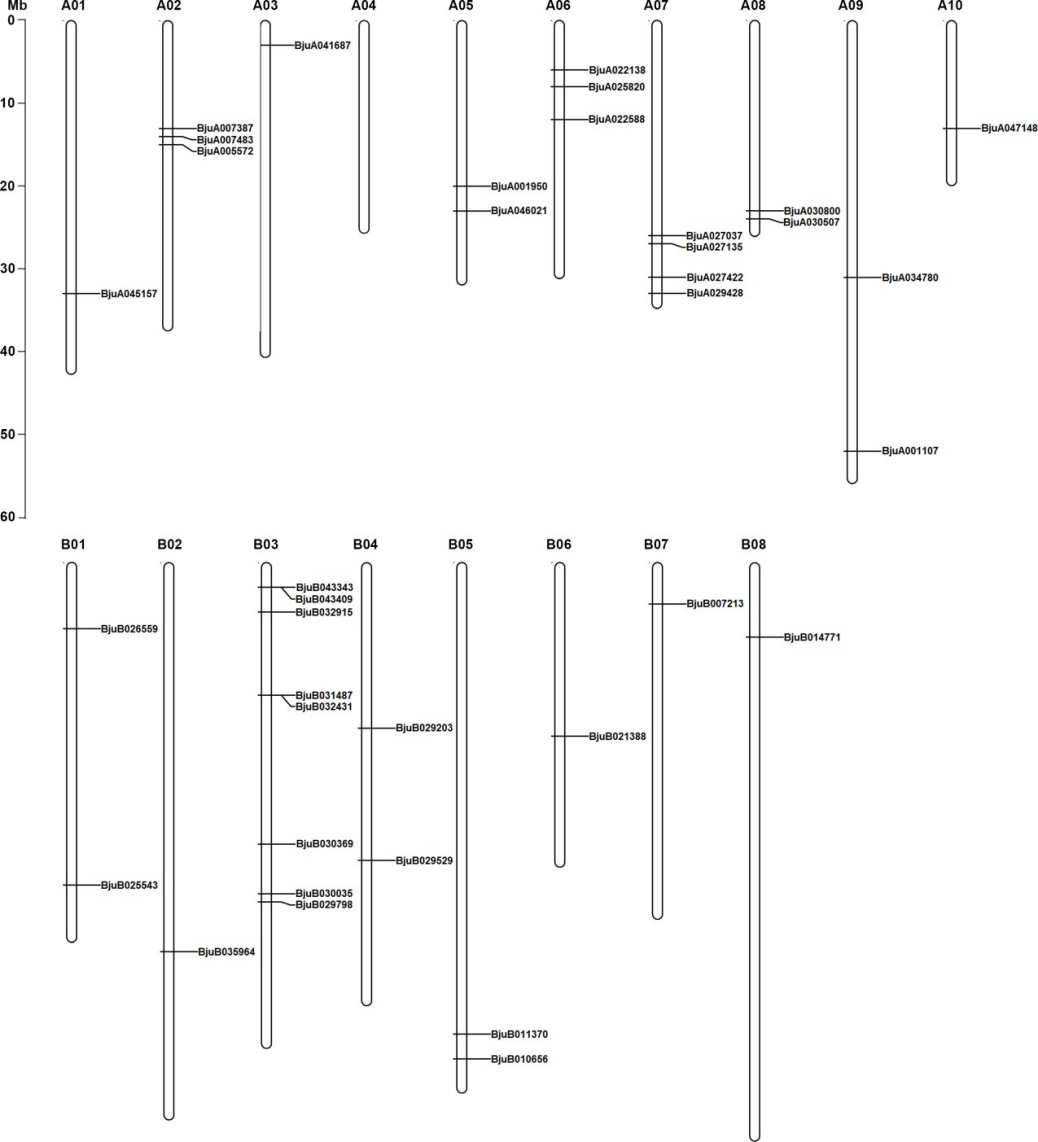
Gene locations of *BjJAZs* in the genome of tuber mustard. Thirty-seven of thirty-eight identified *JAZ* homologous genes were mapped to 17 out of 18 chromosomes. The chromosome name is at the top of each bar. The scale of the chromosome is in millions of bases (Mb).

Thirty-seven of the thirty-eight *BjJAZ* genes were anchored on 18 tuber mustard chromosomes except for chromosome A04 (Fig 2), while *BjuO008948* could not be anchored in the tuber mustard genome. Chromosome B03 had the highest density of *BjJAZ* genes (8 members), and only one *BjJAZ* gene could be found in each of 8 chromosomes (A01, A03, A05, A10, B02, B06, B07 and B08). Four *BjJAZ* genes were located on chromosome A07, and 3 *BjJAZ* genes were located on chromosomes A02 and A06. Each of the chromosomes A08, A09, B04 and B05 contained 2 *BjJAZ* genes (Fig 2). We also analyzed the tandem duplication genes in the whole genome of tuber mustard, and 2588 tandem duplication pairs were identified (S4 File). However, no tandem duplication pairs were found in 38 *BjJAZ* genes.

### Exon/intron structure analysis of *BjJAZs*

**Fig 3:**
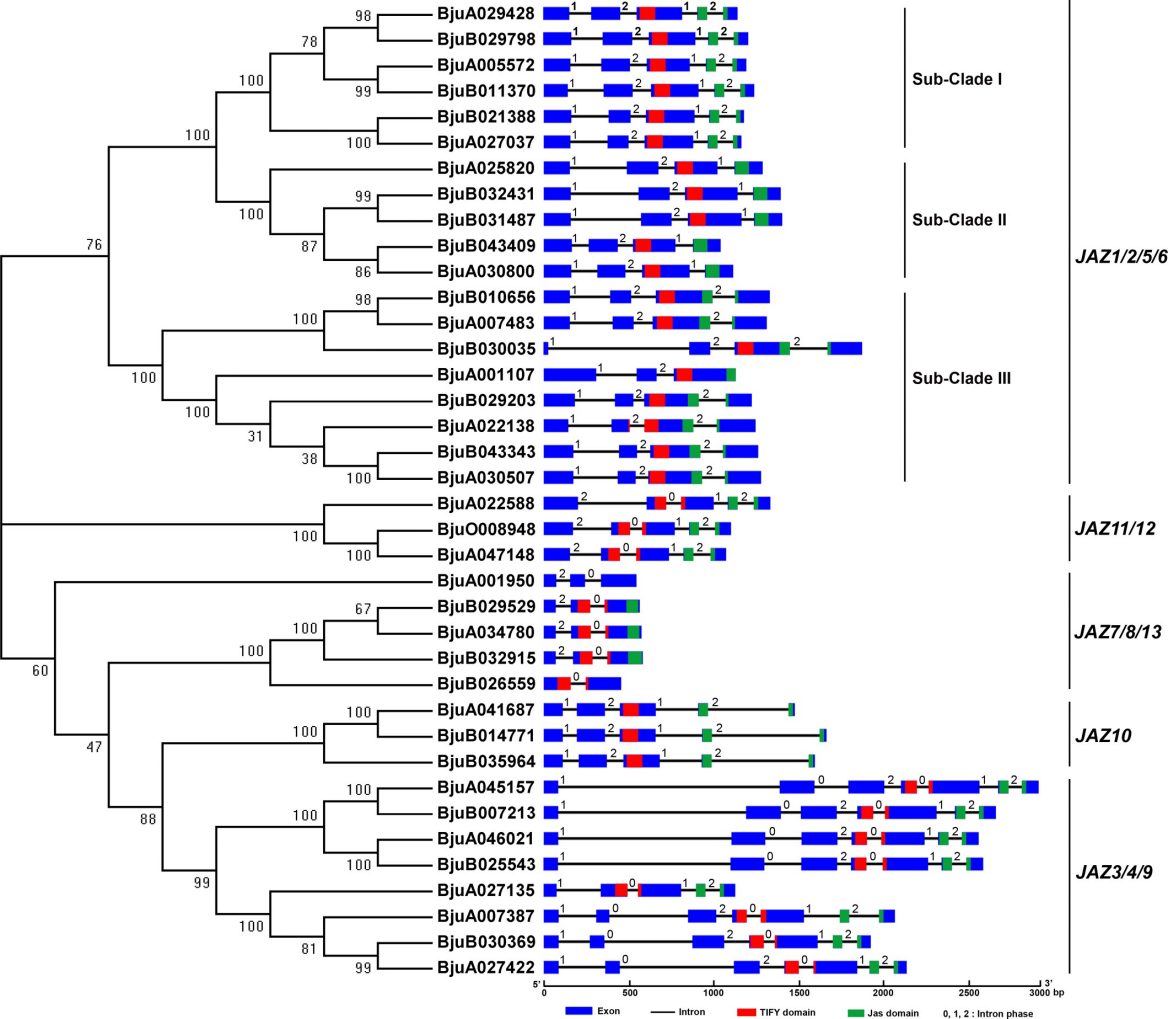
The exon/intron structures of the *BjJAZ* genes. The intron phase was defined as the position of the introns within or between codons. Phase 0: the intron was between codons; phase 1: the intron was between the first and second bases of a codon; phase 2: the intron was between the second and third bases of a codon.

The exon/intron structure of each *BjJAZ* gene was revealed by comparing the CDS with the corresponding genomic sequences. The number of exons in the *BjJAZ* genes varied from 2 (*BjuA001107*) to 7 (*BjJAZ3*/4/9 group genes, except for *BjuA027135*). In *BjJAZ1/2/5/6* group, each of the 6 genes in sub-Clade I contained 5 exons with the intron phase pattern “1,2,1,2”, and each of the 5 genes in sub-Clade II contained 4 exons with the intron phase pattern “1,2,1”. Seven of the eight sub-Clade III genes contained 4 exons with intron phase pattern “1,2,2”, the exception was *BjuA001107*, which contained 3 exons; all of the *BjJAZ11/12* and *BjJAZ10* group genes contained 5 exons, with the intron phase pattern “2,0,1,2” and “1,2,1,2”, respectively; all of the *BjJAZ7/8/13* group genes contained 3 exons with the intron phase pattern “2,0”, except for *BjuA001107* (Fig 3). We also analyzed the intron phase patterns in the TIFY and Jas domains. The results showed that a phase “2” intron inserted in the Jas domain was highly conserved at the 18th amino acid. The *BjJAZ3/4/9*, *BjJAZ7/8* and *BjJAZ11/12* family genes were inserted by a phase “0” intron in the TIYF domain, and no intron insertions were found for the other *BjJAZ* genes (except for *BjuA022138*, which exhibited a phase “2” insertion) (Fig 3). Collectively, most of the genes in the same phylogenetic group (or subclade) exhibited the same exon/intron numbers and intron phase patterns.

### Protein motif analysis of BjJAZs

**Fig 4:**
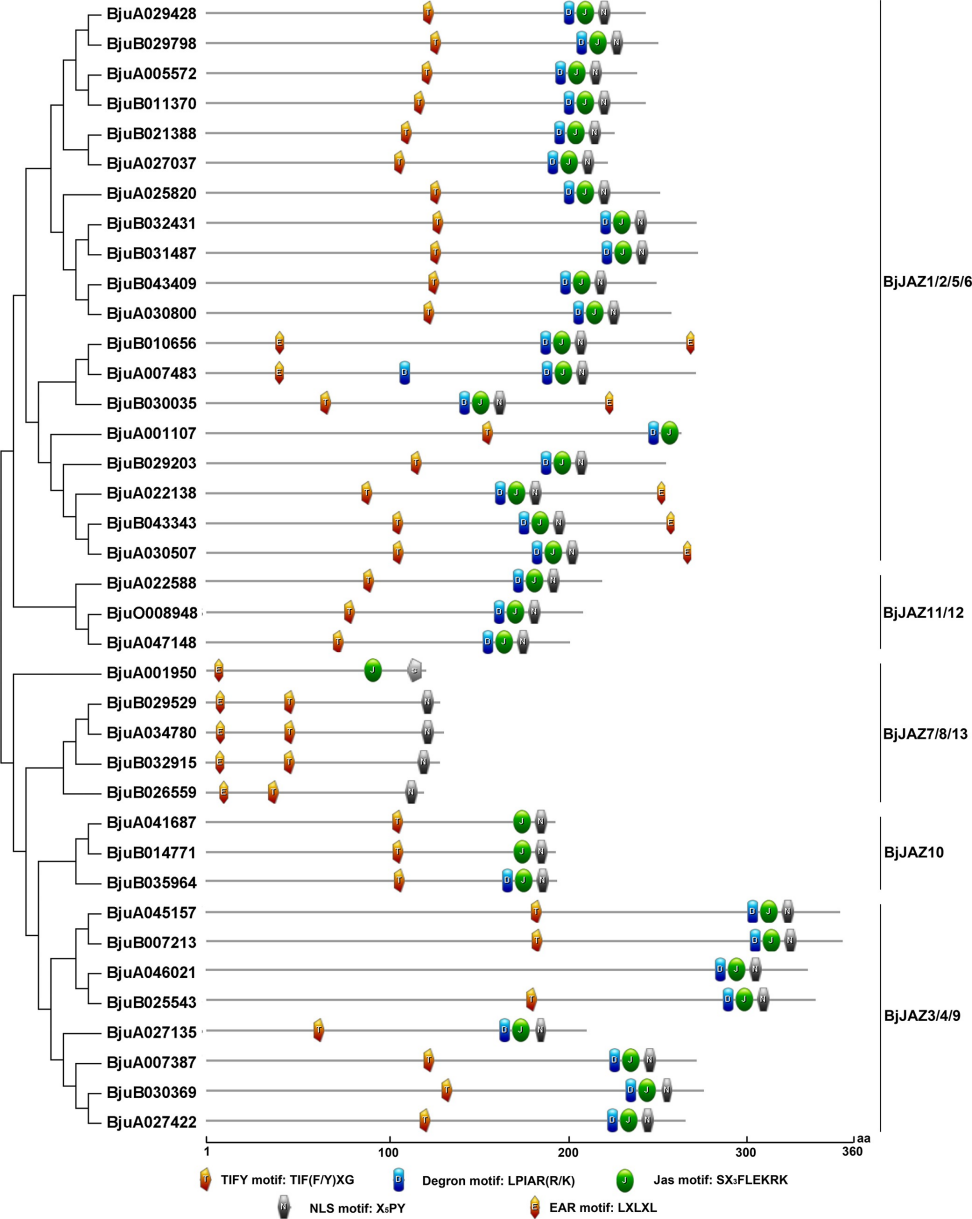
Protein motif prediction for BjJAZs. Five conserved motifs were predicted, and the diagram of the protein motifs was drawn by ExPASy.

**Fig 5:**
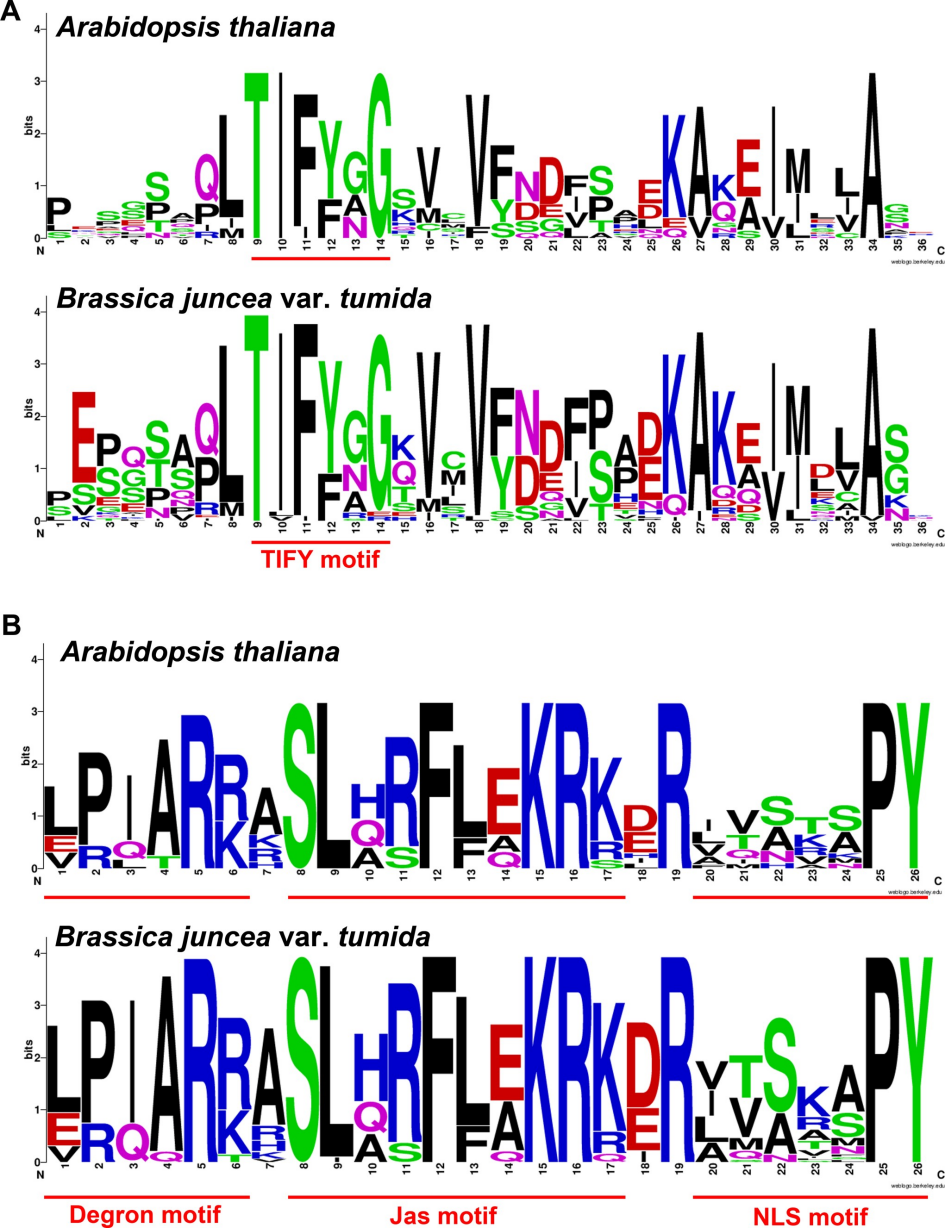
Consensus sequences of the TIFY domain (A) and Jas domain (B) in *Arabidopsis* and tuber mustard. The sequence logos for the TIFY domain and Jas domain were generated with WebLogo 3.

The TIFY domain was previously known as the ZIM domain, which is crucial for interaction with an adapter protein called NINJA. NINJA contains an ethylene-responsive element binding factor associated amphiphilic repression (EAR) motif to recruit TOPLESS. The secondary protein domain prediction results showed that each of the BjJAZ proteins except for BjuA001950 contained one TIFY domain (S1 Fig). Another important domain of the JAZ protein is called the Jas domain (also known as the CCT_2 domain), which is responsible for the COI1-JAZ and MYC2-JAZ interactions. Most of the BjJAZ proteins contained a Jas domain, however, the Jas domain could not be found in BjuB026559 and BjuA001950, and a truncated Jas domain was found in the C-terminus of BjuA001107 (S1 Fig). Several motifs in the TIFY and Jas domains are crucial for the function of JAZ proteins, including the TIFY motif in the TIFY domain; Degron motif, Jas motif, and NLS motif in the Jas domain; and the EAR motif in some JAZ family proteins. The TIFY motif TIF(F/Y)XG is the key component of the TIFY domain, playing important roles in JAZ-NINJA and JAZ-JAZ protein interactions. All the BjJAZ proteins contained a TIFY motif except for BjuA001950 and BjuA046021 (Fig 4). Although no canonical TIFY motif existed in BjuA046021, the TIFY domain was predicted in NCBI-CDD for this protein. The degron motif LPIAR(R/K) is crucial for the COI1-JA-Ile-JAZ complex formation. All the BjJAZ1/2/5/6, BjJAZ3/4/9, and BjJAZ11/12 group proteins contained the degron motif; in contrast, all the BjJAZ7/8/13 group proteins lacked this motif. Interestingly, for the BjJAZ10 group, the degron motif existed in BjuB035964 but not in BjuA041687 and BjuB014771 (Fig 4), and the divergence indicated the different mechanisms by which these BjJAZ10 proteins function in the JA signaling pathway. The Jas motif (SX_3_FLEKRK) is involved in the binding of JAZ to downstream transcription factors. Except for four proteins in BjJAZ7/8/13 group (BjuB026559, BjuB032915, BjuB029529 and BjuA034780), all the other BjJAZ proteins contained this motif. The EAR motif (LxLxL) is related to the interaction with TOPLESS proteins and the resultant repression of JA signaling. The EAR motif existed in 6 protiens of BjJAZ1/2/5/6 group and all of the proteins of BjJAZ7/8/13 group; in particular, there were two EAR motifs in BjuB010656 (Fig 4). Together, the protein motifs were conserved in the same group (except for BjJAZ1/2/5/6); most of the proteins in BjJAZ3/4/9, BjJAZ11/12 and BjJAZ10 groups shared the same motifs. We also analyzed the conservation of the TIFY domain and Jas domain of BjJAZ proteins in tuber mustard and *Arabidopsis*. The results showed that the tuber mustard TIFY domain and Jas domain were well conserved and very similar to those of *Arabidopsis* (Fig 5). The consensus core region of the TIFY motif and Jas motif were conserved between tuber mustard and *Arabidopsis*, and the variation in their flanking regions was also very similar between the two species (Fig 5). Furthermore, to better understand the sequence characteristics of the *BjJAZ* genes, conserved motifs were predicted using MEME based on 38 BjJAZ protein sequences, and 9 motifs (motif 1–9) were identified (S2 Fig). Each of the BjJAZ proteins contained 2 (BjJAZ7/8/13, BjJAZ10 and BjJAZ11/12 proteins) to 9 (part of proteins in BjJAZ3/4/9 group) conserved motifs. The sequence of motif 1 includes the core sequence of Jas motif, and this motif was found in all the BjJAZ proteins (except for BjuA001107); motif 2 includes the core sequence of TIFY motif, and all the BjJAZ proteins contained this motif. Besides that, the distribution of the other motifs was conserved among BjJAZ7/8/13, BjJAZ10 and BjJAZ11/12 groups, but not the other groups (S2 Fig).

### Analysis of transcriptional expression patterns of *BjJAZs* in different tissues

**Fig 6:**
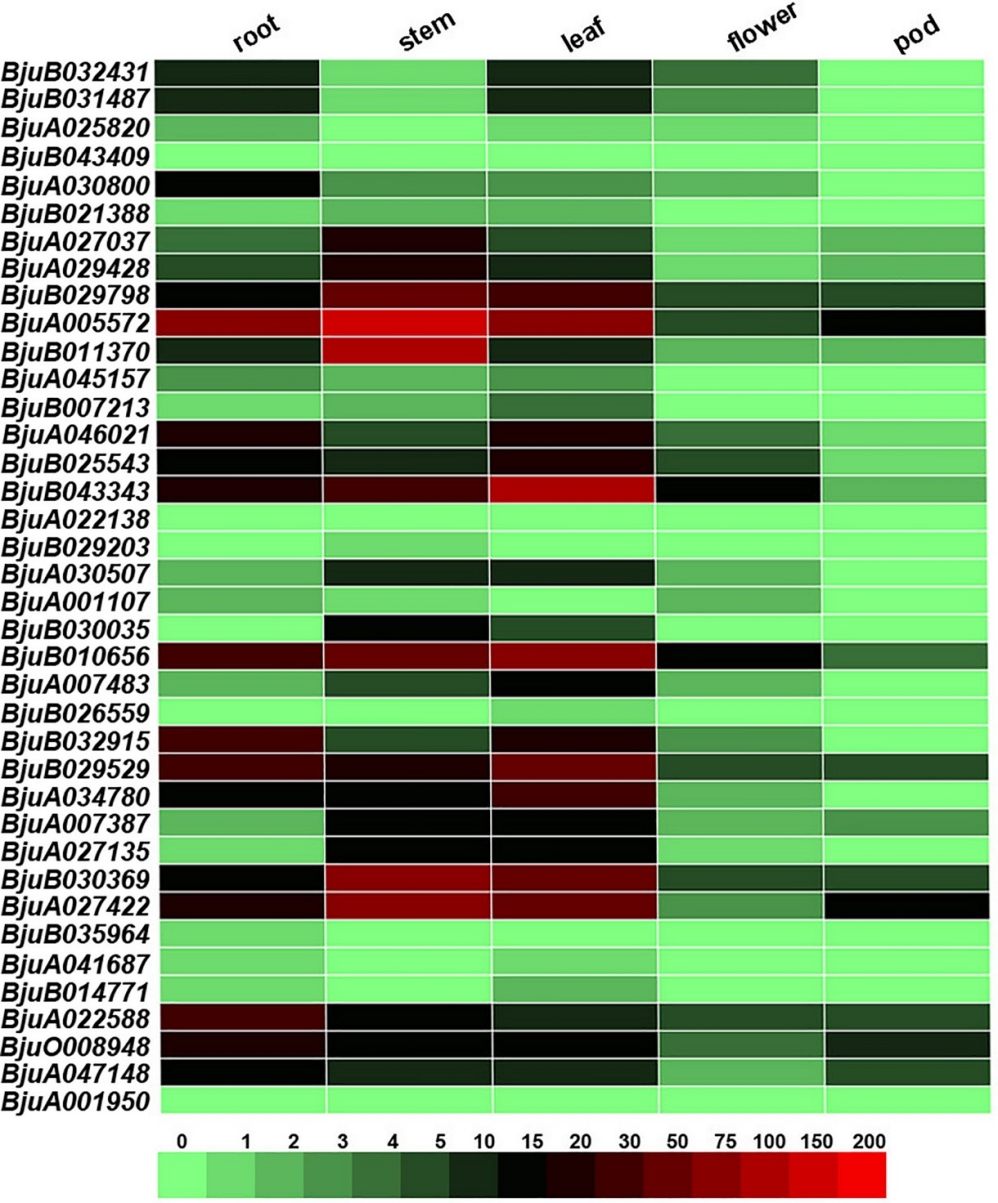
Transcriptional expression patterns of *BjJAZs* in different tissues of tuber mustard. The expression patterns of *BjJAZ* genes were analyzed by qRT-PCR. The Bj18S rRNA gene was used as the internal control. The boxes display the gene expression levels using the 2^−ΔΔCT^ method, and different colors represent the different expression levels of each gene.

To investigate the gene expression patterns of *BjJAZs*, different tissues (root, stem, leaf, flower and pod) of tuber mustard were collected and qRT-PCR assays were performed to detect the expression levels of each *BjJAZ* gene in different organs. The results showed that the genes *BjuB043409*, *BjuA022138*, *BjuB029203*, *BjuB026559*, *BjuB035964*, *BjuA041687*, *BjuB014771* and *BjuA001950* were expressed at low levels in most organs, while *BjuA005572*, *BjuB010656*, *BjuB032915*, *BjuB029529*, *BjuA027422*, *BjuA022588*, *BjuO008948* were expressed at high levels in roots; *BjuB029798*, *BjuA005572*, *BjuB011370*, *BjuB043343*, *BjuB010656*, *BjuB030369*, *BjuA027422* were highly expressed in stems; and *BjuB029798*, *BjuA005572*, *BjuB043343*, *BjuB010656*, *BjuB029529*, *BjuA034780*, *BjuB030369*, *BjuA027422* were highly expressed in leaves. All the *BjJAZ* genes except for *BjuA005572*, *BjuB043343*, *BjuB010656* and *BjuA027422* were expressed at low levels in flowers (except for *BjuB043343* and *BjuB010656*) and pods (except for *BjuA005572* and *BjuA027422*). *BjuA030800*, *BjuA022588* and *BjuA047148* were expressed more strongly in the roots than in the other organs. *BjuA027037*, *BjuB011370* and *BjuB030035* showed higher expression levels in stems than in the other organs (Fig 6). *BjuB043343* and *BjuA007483* showed relatively higher expression levels in leaves than in the other organs (Fig 6). The results suggested that these *BjJAZ* genes with tissue-specific expression might play roles in the regulation of the relative organs growth and development.

### Analysis of transcriptional expression patterns of *BjJAZs* under *Plasmodiophora brassicae* treatment

**Fig 7:**
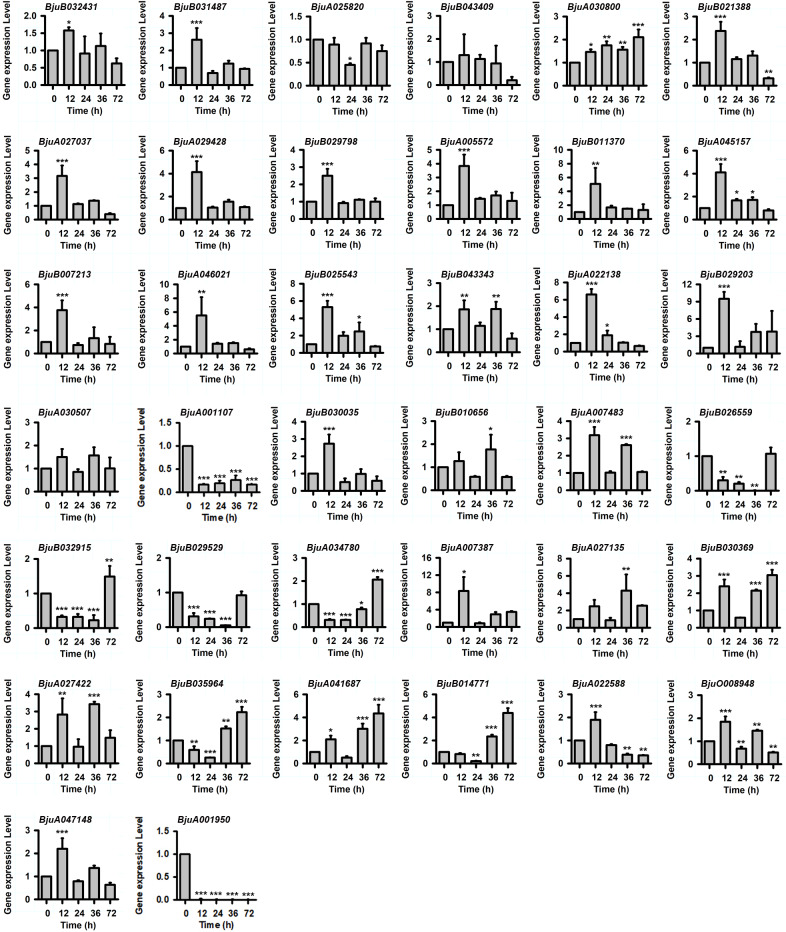
Expression patterns of *BjJAZ* genes under treatment with the pathogen *Plasmodiophora brassicae*. The expression patterns of *BjJAZ* homologous genes were analyzed by qRT-PCR. The Bj18S rRNA gene was used as the internal control. Three independent biological repeats were performed, and all data points are the means of three biological replicates ± standard errors (SEs). Significant differences: *, *p* < 0.05.; **, *p* < 0.01.; ***, and *p* < 0.001.

*P*. *brassicae*, the main pathogen of tuber mustard, is a severe pathogen that frequently attacks tuber mustard seedlings and causes substantial production losses. To investigate the functions of the *BjJAZ* genes in plant resistance to the pathogen *P*. *brassicae*, the transcriptional expression levels of these genes were assessed in the tuber mustard roots treated with *P*. *brassicae*. The results showed that *BjuB031487*, *BjuB021388*, *BjuA027037*, *BjuA029428*, *BjuB029798*, *BjuA005572*, *BjuA045157*, *BjuB007213*, *BjuB025543*, *BjuA022138*, *BjuB030035*, *BjuA022588* and *BjuA047148* were upregulated at 12 h after *P*. *brassicae* treatment compared to the control, and then the expression of these genes decreased (Fig 7). *BjuA007483* was mainly induced by *P*. *brassicae* at 12 h and 36 h, while all three *BjJAZ10* group genes were mainly induced at 36 and 72 h (Fig 7). In contrast, the expression levels of *BjuB026559*, *BjuB032915*, *BjuB029529* and *BjuA034780* were significantly decreased after 12, 24, and 36 h of *P*. *brassicae* treatment, and the expression of these genes decreased at 72 h. *BjuA001107* and *BjuA001950* were significantly repressed by *P*. *brassicae* from 12 to 72 h. These results indicate that these genes might be involved in the tuber mustard response to *P*. *brassicae* stress.

### Analysis of transcriptional expression patterns of *BjJAZs* under salt-stress treatment

**Fig 8:**
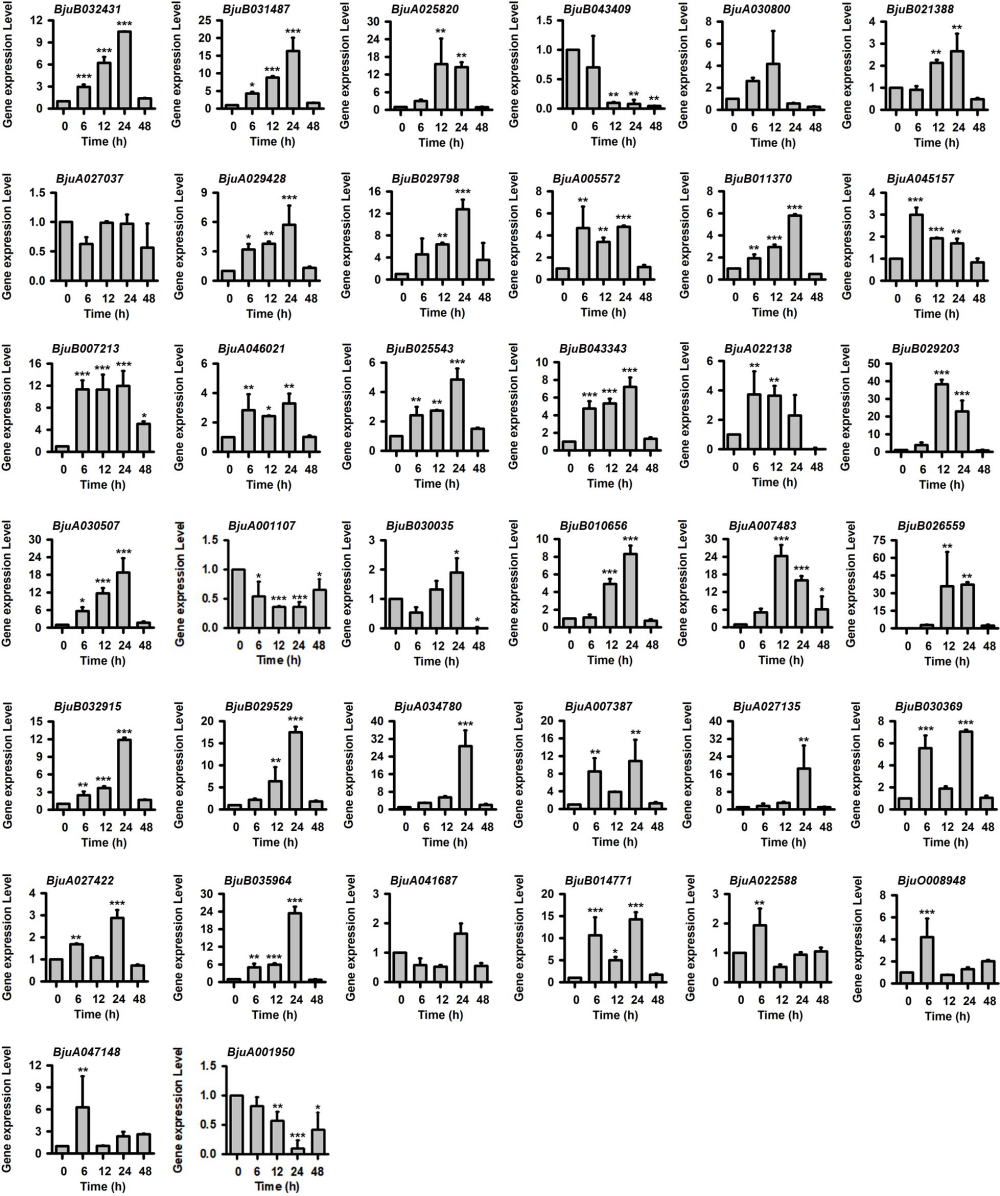
Expression patterns of *BjJAZ* genes under salt stress treatment. The expression patterns of *BjJAZ* homologous genes were analyzed by qRT-PCR. Bj18S rRNA gene was used as the internal control. Three independent biological repeats were performed, and all data points were the means of three biological replicates ± standard error (SE). Significant differences: *, *p* < 0.05.; **, *p* < 0.01.; and ***, *p* < 0.001.

Tuber mustard is frequently attacked by salt stress during plant growth in the field. To identify the *BjJAZ* genes involved in the plant response to salt stress, 2‐week‐old seedlings were treated with 200 mM NaCl for 0, 6, 12, 24 and 48 h, and the expression levels of the *BjJAZ* family genes were evaluated by qRT-PCR. The results showed that nearly no *BjJAZ* gene was downregulated by salt-stress treatment; in contrast, the expression levels of some *BjJAZ* genes increased at different time points after 200 mM NaCl treatment. The genes *BjuB032431*, *BjuB007213* and *BjuB043343* were continuously induced from 6 to 24 h; *BjuB031487*, *BjuB011370*, *BjuA030507*, *BjuB010656*, *BjuA007483*, *BjuB032915* and *BjuB035964* were mainly upregulated at 12 and 24 h. Some genes were mainly induced by salt stress at 24 h after treatment, such as *BjuA029428*, *BjuB029798*, *BjuA005572*, *BjuB025543*, *BjuB029529*, *BjuA034780* and *BjuA027422* (Fig 8). In contrast, the expression level of *BjuA001107* was significantly decreased at 12 to 24 h, and the expression of *BjuA001950* was decreased at 24 h. The results indicate that these genes might play roles in the tuber mustard response to salt stress.

## Discussion

Tuber mustard is the raw material of Fuling pickle, which has great economic value. The mechanisms involved in the regulation of tuber mustard growth and development and responses to biotic and abiotic stress remain to be further investigated. Although it has been reported that some *JAZ* genes play roles in plant growth, development and responses to abiotic stress [25,26], the identities and functions of tuber mustard *JAZ* genes remain unknown. Here, we identified the *BjJAZ* genes in the whole genome of tuber mustard and investigated their expression patterns during growth and development and their possible roles in plant responses to salt and pathogen stress.

According to the results of gene sequence identity and protein domain conservation analyses, 38 *BjJAZ* genes were identified in the genome of tuber mustard, and the number of *BjJAZs* is more than double that in *Arabidopsis* (13), rice (15) and *Brachypodium distachyon* (15) [27–29]. The reason might be that tuber mustard is an allotetraploid species formed by hybridization between the diploid ancestors of *Brassica rapa* and *Brassica nigra* with subsequent genome duplication [30]; genome duplication is also found in moso bamboo, the genome of which contains 18 *PeJAZ* genes [31]. Gene duplication was also found among *JAZ* genes in apple, the genome of which contained 18 *MdJAZ* genes [32]. In a previous study, the origin and evolution of TIFY transcription factors were investigated using 14 genomes from all kingdoms of life, and the results indicated that whole genome duplication and tandem duplication contributed to the expansion of *TIFY* family genes [33]. However, no tandem duplication pairs were found in 38 *BjJAZ* genes. Whole genome duplication might be the main factors resulting in the expansion of *BjJAZ* family genes.

Exon/intron structural divergence plays important roles in the evolution of multiple gene families. Most of the *BjJAZ* genes have the same number of exons comparing with the relative *JAZ* genes in *Arabidopsis*. However, the divergence of exon number was also found among *BjJAZ* and *AtJAZ* genes. For example, although five *BjJAZ* genes (*BjuA025820*, *BjuB032431*, *BjuB031487*, *BjuB043409*, *BjuA030800*) and *AtJAZ1* formed a single orthologous cluster (S3 File), they had different number of exons: 4 exons were found in the five *BjJAZ* genes, but only 2 in AtJAZ1 (Fig 3). The exon/intron gene structure reflected the diversification and evolution of these genes through gain/loss and insertion/deletions, and the diversity of exon/intron structure between different groups may be related to their functional evolution. JAZ family proteins are defined by the highly conserved TIFY domain and Jas domain. However, the recently identified JAZ13 lacks a TIFY domain. It was reported that the *Arabidopsis* JAZ13 protein together with its orthologs in Brassicaceae species contained a “NAFXXG” motif in place of the highly conserved “TIF(F/Y)XG” motif [28]. Similar to the JAZ13 protein in *Arabidopsis*, BjuA001950 also contains a divergent “NAFYSG” sequence instead of a canonical TIFY motif (S1 Fig). In *Arabidopsis*, the EAR motif specifically exists in JAZ5, JAZ6, JAZ7 and JAZ8 [34]. In tuber mustard, EAR motifs were also found insome proteins of BjJAZ1/2/5/6 and BjJAZ7/8/13 group (Fig 4). Taken together, the results show that the common motifs are shared by tuber mustard and *Arabidopsis*, suggesting the conserved evolutionary processes between the two species.

Gene expression patterns often have a connection with gene function, and some *BjJAZ* genes with tissue-specific expression were identified in tuber mustard, which suggested their roles in specific tissues. In wheat, *TaJAZ5* was observably highly expressed in leaf tissues, and *TaJAZ6* was specifically expressed in the roots [35]. In contrast, *BjuA030800* and *BjuA022588* were expressed more strongly in the roots than in the other organs, suggesting the different roles of these genes in the two species (Fig 6).

*P*. *brassicae* is the main pathogen during tuber mustard growth and development, and a number of *BjJAZ* genes are induced by this pathogen, suggesting the common roles of JA signaling in plant responses to this pathogen. The roles of *JAZ* genes in plant resistance to pathogens have been reported in other crops. For example, transgenic lines overexpressing apple *MdJAZ2* were less susceptible to *P*. *syringae* pv. *tomato DC3000* than wild-type seedlings of *Arabidopsis* [36]. In *Arabidopsis*, all *AtJAZ* genes, except for *AtJAZ3*, *AtJAZ4*, *AtJAZ11* and *AtJAZ12*, were induced by infection with the pathogen *P*. *syringae DC3000* from 24 to 48 h [14]. In accordance with this finding, most *BjJAZ* genes were induced by treatment with the pathogen *P*. *brassicae* (Fig 7), suggesting similar roles in the plant response to pathogen attacks. However, four *BjJAZ* genes (BjuB026559, BjuB032915, BjuB029529, BjuA034780) were significantly repressed by treatment with this pathogen from 12 to 36 h (Fig 7), indicating their different roles in the tuber mustard response to *P*. *brassicae*. In rice, although the expression level of *OsJAZ8* was stable after the seedlings were treated with the pathogen *X*. *oryzae* pv. *oryzae* (*Xoo*), the overexpression of Jas domain-truncated *OsJAZ8* confirmed that this gene negatively regulated JA-induced resistance to *Xoo* in rice [37]. In tuber mustard, *BjuA034780* and *BjuB029529* were identified as co-orthologs of *AtJAZ8* (S3 File), and they were significantly repressed by the pathogen *P*. *brassicae*, which merits further study.

Salinity is the main abiotic stress factor for tuber mustard in the planting area. According to the expression patterns of *BjJAZ* genes under salt-stress treatment, the salt stress responsive *BjJAZ* genes might play roles in tuber mustard tolerance to salinity. Similarly, in *Gossypium hirsutum*, 14 of 30 *GhJAZ* genes were significantly induced by salt-stress treatment from 3 to 6 h [15]. In *Glycine soja*, the transcriptional level of *GsJAZ2* increased from 1 to 6 h of 200 mM NaCl treatment in the roots, and overexpression of *GsJAZ2* enhanced the salt tolerance of transgenic *Arabidopsis* seedlings [38]. In addition, overexpression of the apple *MdJAZ2* gene also improved plant tolerance to NaCl stress treatment in transgenic *Arabidopsis* [36]. In tuber mustard, three *AtJAZ2* orthologous genes (*BjuA029428*, *BjuA005572*, *BjuB011370*) were significantly induced by 200 mM NaCl (Fig 8, S3 File), suggesting similar roles for these genes in plant tolerance to salt stress. In wheat, several *TaJAZ* genes, such as *TaJAZ1*, *TaJAZ2*, *TaJAZ5*, *TaJAZ9* and *TaJAZ10*, were induced by high-salinity treatment [35]. The qRT-PCR results also showed that the expression of a number of *BjJAZ1/2/5/6* and *BjJAZ10* group genes was induced by salt treatment, indicating that the functions of the *JAZ* genes in the plant response to salinity are probably conserved between the two species. In moss, the expression level of *PnJAZ1* was increased after NaCl treatment, and both the transgenic lines of *Arabidopsis* and *Physcomitrella* overexpressing *PnJAZ1* showed increased tolerance to salt stress [39], suggesting the conserved function of *JAZ1* in the plant response to salt stress in lower and higher plant species. Some divergent expression patterns of *JAZ* genes were also found in other species. For example, in *Solanum lycopersicum*, *SlJAZ3* and *SlJAZ10* were consistently induced by salt treatment in leaves but downregulated in roots [40]. The different expression patterns of *JAZ*-orthologous genes in different species indicated the complex roles of JAZ proteins in the regulation of JA signaling. Further study of the detailed functions of biotic and abiotic stress-responsive *BjJAZ* genes will shed light on the mechanism of resistance of tuber mustard to these forms of stress.

## Supporting information

S1 Table Primers used for qRT-PCR in this study. (DOCX)

S1 Fig Sequence alignment of the TIFY domain and Jas domain of the BjJAZ proteins. (TIF)

S2 Fig Conserved motifs of 38 BjJAZ proteins predicted by MEME software. (TIF)

S1 File Peptide sequences of JAZ proteins in the genome of *Arabidopsis thaliana*. (DOCX)

S2 File Peptide sequences of JAZ proteins in the genome of *Braassica juncea* var. *tumida*. (DOCX)

S3 File Orthologous gene clusters analysis between BjJAZs and AtJAZs. (XLSX)

S4 File Tandem duplication pairs in the genome of *Brassica juncea* var. *tumida*. (XLSX)
