# Supplementary material for: Genome-wide identification and expression analysis of jasmonate ZIM domain gene family in tuber mustard (Brassica juncea var. tumida)
Source: PLoS One. 2020 Jun 16;15(6):e0234738. doi: 10.1371/journal.pone.0234738 (PMC7297370; doi:10.1371/journal.pone.0234738)
Supplement: S1 Table — (DOCX) [file pone.0234738.s001.docx]

**Supplemental Table 1 Primers used for qRT-PCR in this study**

| **Gene ID** | **Primer Sequence (Forward)** | **Primer Sequence (Reverse)** |
| --- | --- | --- |
| *BjuB032431* | GCTTCACTCCATCGGTTCTTG | TTGGCTGGATCGTTTAGTTGAT |
| *BjuB031487* | CGCCGCTACTCGGAGATTT | CGTGAGATGCCGTTGACCT |
| *BjuA025820* | CAAGACCCAAAGCAAACCTCC | TCGCCAAGAACCGATGAAG |
| *BjuB043409* | GCTTGCGAACTTCCTATTGCT | CATGATGTGGCGTTGTCTCC |
| *BjuA030800* | CTCTGATTCCGCCGCTACTC | GATCTCCAAAGCTGCCGTTC |
| *BjuB021388* | CTGCCGAGTGTTGGGAGTT | GCATGTCATTGTCATCACGAAGT |
| *BjuA027037* | AGCCAATCCAGACCAACACG | CGGTCCCTTTGATGTAATCCTATC |
| *BjuA029428* | CGGCGGTCAAGTTATGGTC | CTGGGGTAGTGGAAGCAATCT |
| *BjuB029798* | TGATGATTTTCCTGCTGAGATAGC | CGAAGAACTGGATTTGGGGTA |
| *BjuA005572* | GTGTCACGTCAGCCAACGAT | CAGCGGAAGAGGAAGAAGAAA |
| *BjuB011370* | CATGGTTCCTCGCCAGACA | GACTCCACCTTCACGGATTTAG |
| *BjuA045157* | AGTTCTGTTCAGGGAGTTAGGATG | CCAAAGGTTGCGAGTTTACG |
| *BjuB007213* | CCCAAGAAGACAGACATAGAAACA | AACATCCTAACTCCCTGAACAGAA |
| *BjuA046021* | TGAAAGAGGAAACAAGCGAAAG | AACTGAGAAGAAGCAGCAGAGC |
| *BjuB025543* | CATCATGCTCGTGCCTCTGT | GCCTGTCGTTGCTTTTACTGCT |
| *BjuB043343* | GGAACGTATAGCACGTAGAGCG | GGGAGGATAGTGATGACGACCA |
| *BjuA022138* | AGATGTAGTCTGCTCAGCCGTTAC | CCGAATCCGCCTTATGTTGT |
| *BjuB029203* | AACAAAATCATGTCGTGGAACG | GGAGGATAACTTGGTATGGAGCA |
| *BjuA030507* | GAAGGAGAAGGGTAGTTTCGGTA | CCTAAGACATTAAGGGCTTTGAGT |
| *BjuA001107* | GCAAAGGCACTCGGACCG | CTGCCTGGTGGACAGGAGTA |
| *BjuB030035* | TCCTCCCAAGCCACAAATG | AACACCGTCCTTCTTCTTCCTC |
| *BjuB010656* | TTTGCTAAACGAAAAGACAGGG | TGGCTTGGGTGGAAGATGA |
| *BjuA007483* | AGACAAAGCAAAGGAGATAATGGC | TGGCTCGTTCAGATCAGGAAT |
| *BjuB026559* | ATCCTCCGATAATCCCAAACA | GTAAGGGCAAGAAGCCTGAAT |
| *BjuB032915* | AACAGGTTGACCCGATTACATC | TCGTCGTGATTGCTGATGGT |
| *BjuB029529* | CAGCTTCCGAATCCAAAGG | GTTGATGGTAAGGTGAAGTGGC |
| *BjuA034780* | ACTGCGACTTGGAACTTCGTC | TTCCCAGAGCTTCTTGATTCTACC |
| *BjuA007387* | CGTGTCGGAGAACGAGTATTG | AACTGGTCCCCGCTGGTAAG |
| *BjuA027135* | ATTCCATAACGCTGCTACTGCT | ACCAACGGGTCTGTCTCCAC |
| *BjuB030369* | GGTTTGAGCGACAAGCAGTATC | AAGAGTTTTGCCTTTCCCCAT |
| *BjuA027422* | GACAAATGCAATGACCATGACG | GCCAGCGATGCTTTACGAG |
| *BjuB035964* | TTGACCGCCGTCGTAGTTT | CGGAAGGGTTAGCACCAGAAG |
| *BjuA041687* | CCATGCAAACGGAACATAGC | AGACACTTGGTAAACGGAGACG |
| *BjuB014771* | TGGAACCGTCTCCGTTTACC | TCTTTGGTGCTGTTTCCTTGAC |
| *BjuA022588* | CAAAGAACTCTACAAGCGTGAAAC | GCAAAACGGAATTGGGTTAA |
| *BjuO008948* | CGGAAAAGGTGCTGAGAAACA | TGGAATCCCATTGAAGACGC |
| *BjuA047148* | CGGAGAAGGTGCTGAGAAAGA | TGGAATCCCATCAAAGACGC |
| *BjuA001950* | CTTCACTTCAATCTTGTCGCC | CTCGTACTCTCCCACTATAGAATGC |
| *BjuA046942* (*Bj18 s rRNA*) | TCTGACGCAAGCACAACTA | TCTTCTCAGCTTTAGCCATT |
