# Supplementary figures and images for: Genome-wide identification and expression analysis of jasmonate ZIM domain gene family in tuber mustard (Brassica juncea var. tumida)
Source: PLoS One. 2020 Jun 16;15(6):e0234738. doi: 10.1371/journal.pone.0234738 (PMC7297370; doi:10.1371/journal.pone.0234738)

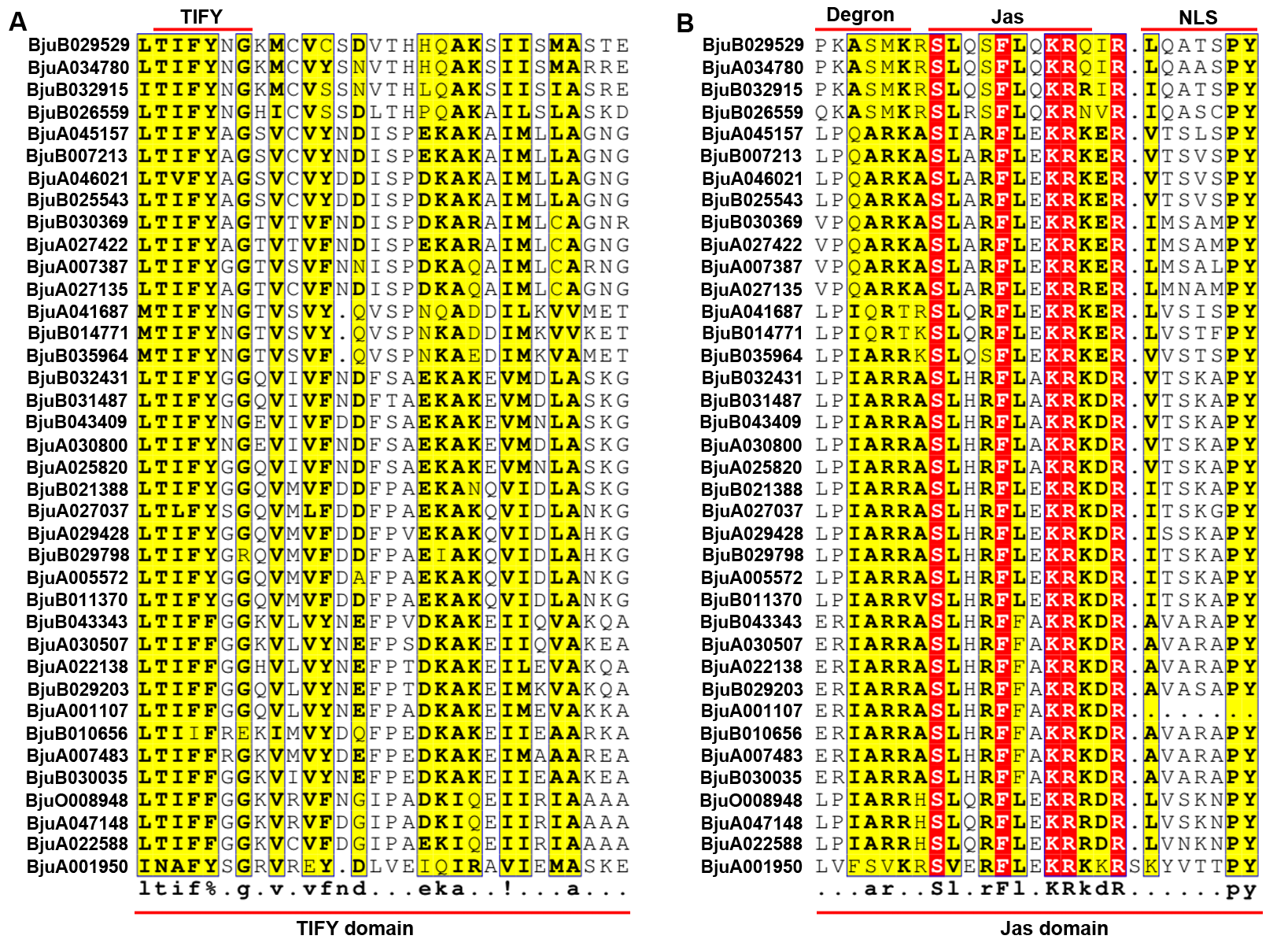

Supplement: S1 Fig — (TIF) [file pone.0234738.s002.tif]

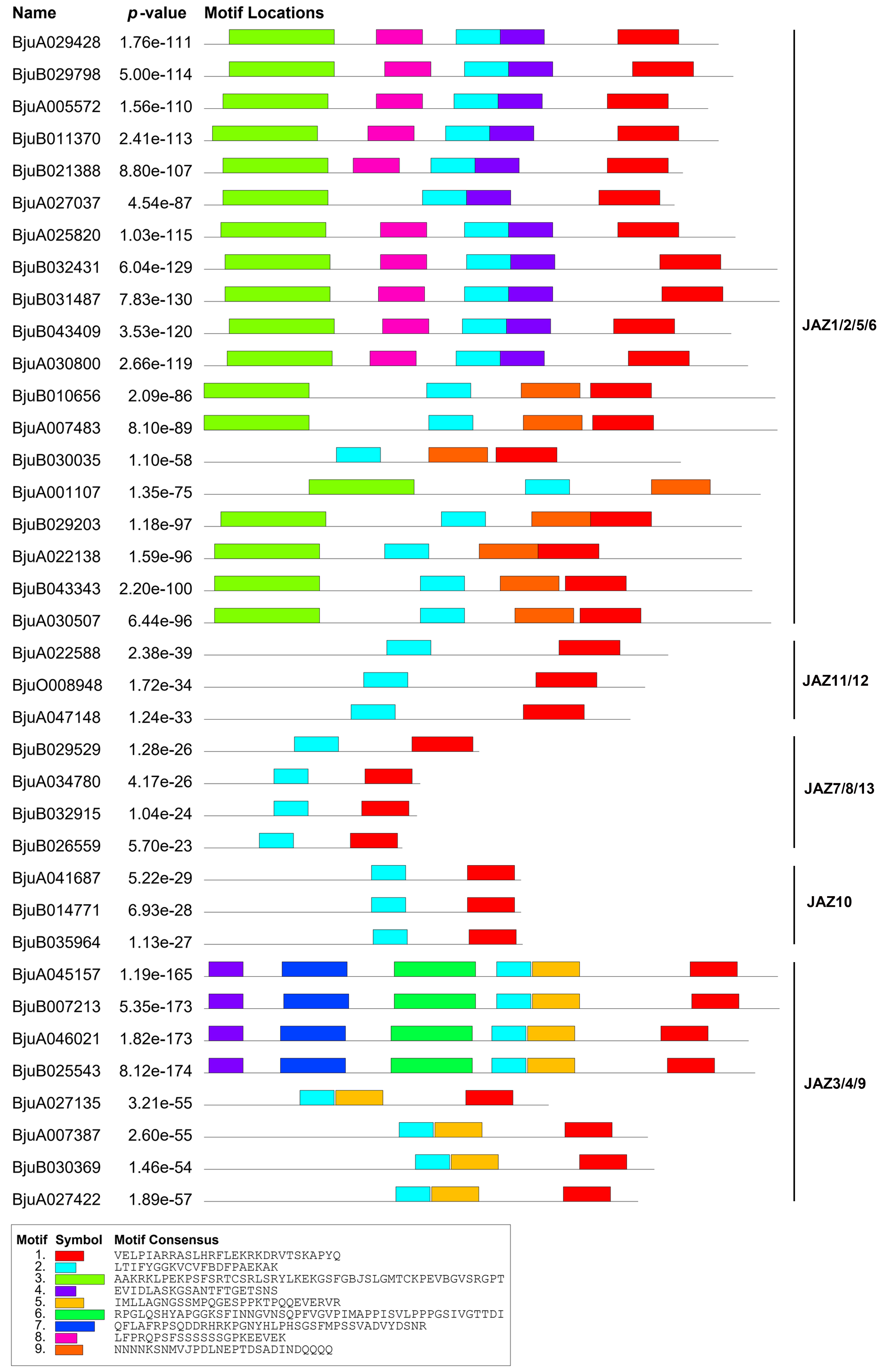

Supplement: S2 Fig — (TIF) [file pone.0234738.s003.tif]
