# Supplementary material for: Genome-wide identification and expression analysis of jasmonate ZIM domain gene family in tuber mustard (Brassica juncea var. tumida)
Source: PLoS One. 2020 Jun 16;15(6):e0234738. doi: 10.1371/journal.pone.0234738 (PMC7297370; doi:10.1371/journal.pone.0234738)
Supplement: S1 File — (DOCX) [file pone.0234738.s004.docx]

**Supporting information file 1:** the peptide sequences of JAZ proteins in the genome of *Arabidopsis*.

>AtJAZ1（At1G19180）

MSSSMECSEFVGSRRFTGKKPSFSQTCSRLSQYLKENGSFGDLSLGMACKPDVNGTLGNSRQPTTTMSLFPCEASNMDSMVQDVKPTNLFPRQPSFSSSSSSLPKEDVLKMTQTTRSVKPESQTAPLTIFYAGQVIVFNDFSAEKAKEVINLASKGTANSLAKNQTDIRSNIATIANQVPHPRKTTTQEPIQSSPTPLTELPIARRASLHRFLEKRKDRVTSKAPYQLCDPAKASSNPQTTGNMSWLGLAAEI

>AtJAZ2（At1G74950）

MSSFSAECWDFSGRKPSFSQTCTRLSRYLKEKGSFGDLSLGMTCKPDVNGGSRQPTMMNLFPCEASGMDSSAGQEDIKPKTMFPRQSSFSSSSSSGTKEDVQMIKETTKSVKPESQSAPLTIFYGGRVMVFDDFSAEKAKEVIDLANKGSAKSFTCFTAEVNNNHSAYSQKEIASSPNPVCSPAKTAAQEPIQPNPASLACELPIARRASLHRFLEKRKDRITSKAPYQIDGSAEASSKPTNPAWLSSR

>AtJAZ3（At3G17860）

MERDFLGLGSKNSPITVKEETSESSRDSAPNRGMNWSFSNKVSASSSQFLSFRPTQEDRHRKSGNYHLPHSGSFMPSSVADVYDSTRKAPYSSVQGVRMFPNSNQHEETNAVSMSMPGFQSHHYAPGGRSFMNNNNNSQPLVGVPIMAPPISILPPPGSIVGTTDIRSSSKPIGSPAQLTIFYAGSVCVYDDISPEKAKAIMLLAGNGSSMPQVFSPPQTHQQVVHHTRASVDSSAMPPSFMPTISYLSPEAGSSTNGLGATKATRGLTSTYHNNQANGSNINCPVPVSCSTNVMAPTVALPLARKASLARFLEKRKERVTSVSPYCLDKKSSTDCRRSMSECISSSLSSAT

>AtJAZ4（At1G48500）

MERDFLGLGSKLSPITVKEETNEDSAPSRGMMDWSFSSKVGSGPQFLSFGTSQQETRVNTVNDHLLSSAAMDQNQRTYFSSLQEDRVFPGSSQQDQTTITVSMSEPNYINSFINHQHLGGSPIMAPPVSVFPAPTTIRSSSKPLPPQLTIFYAGSVLVYQDIAPEKAQAIMLLAGNGPHAKPVSQPKPQKLVHHSLPTTDPPTMPPSFLPSISYIVSETRSSGSNGVTGLGPTKTKASLASTRNNQTAAFSMAPTVGLPQTRKASLARFLEKRKERVINVSPYYVDNKSSIDCRTLMSECVSCPPAHHLH

>AtJAZ5（At1G17380）

MSSSNENAKAQAPEKSDFTRRCSLLSRYLKEKGSFGNIDLGLYRKPDSSLALPGKFDPPGKQNAMHKAGHSKGEPSTSSGGKVKDVADLSESQPGSSQLTIFFGGKVLVYNEFPVDKAKEIMEVAKQAKPVTEINIQTPINDENNNNKSSMVLPDLNEPTDNNHLTKEQQQQQEQNQIVERIARRASLHRFFAKRKDRAVARAPYQVNQNAGHHRYPPKPEIVTGQPLEAGQSSQRPPDNAIGQTMAHIKSDGDKDDIMKIEEGQSSKDLDLRL

>AtJAZ6（At1G72450）

MSTGQAPEKSNFSQRCSLLSRYLKEKGSFGNINMGLARKSDLELAGKFDLKGQQNVIKKVETSETRPFKLIQKFSIGEASTSTEDKAIYIDLSEPAKVAPESGNSQLTIFFGGKVMVFNEFPEDKAKEIMEVAKEANHVAVDSKNSQSHMNLDKSNVVIPDLNEPTSSGNNEDQETGQQHQVVERIARRASLHRFFAKRKDRAVARAPYQVNQHGSHLPPKPEMVAPSIKSGQSSQHIATPPKPKAHNHMPMEVDKKEGQSSKNLELKL

>AtJAZ7（At2G34600）

MIIIIKNCDKPLLNFKEMEMQTKCDLELRLLTSSYDSDFHSSLDESSSSEISQPKQESQILTIFYNGHMCVSSDLTHLEANAILSLASRDVEEKSLSLRSSDGSDPPTIPNNSTRFHYQKASMKRSLHSFLQKRSLRIQATSPYHRYR

>AtJAZ8（At1G30135）

MKLQQNCDLELRLFPTSYDSDSSDTTSVVESTSSGNPQPNEESQRITIFYNGKMCFSSDVTHLQARSIISIASREMKTKSSSNGSDPPNKSTSFHHNQLPNPKASMKKSLQSFLQKRKIRIQATSPYHSRR

>AtJAZ9（At1G70700）

MERDFLGLSDKQYLSNNVKHEVNDDAVEERGLSTKAAREWGKSKVFATSSFMPSSDFQEAKAFPGAYQWGSVSAANVFRRCQFGGAFQNATPLLLGGSVPLPTHPSLVPRVASSGSSPQLTIFYGGTISVFNDISPDKAQAIMLCAGNGLKGETGDSKPVREAERMYGKQIHNTAATSSSSATHTDNFSRCRDTPVAATNAMSMIESFNAAPRNMIPSVPQARKASLARFLEKRKERLMSAMPYKKMLLDLSTGESSGMNYSSTSPT

>AtJAZ10（At5G13220）

MSKATIELDFLGLEKKQTNNAPKPKFQKFLDRRRSFRDIQGAISKIDPEIIKSLLASTGNNSDSSAKSRSVPSTPREDQPQIPISPVHASLARSSTELVSGTVPMTIFYNGSVSVFQVSRNKAGEIMKVANEAASKKDESSMETDLSVILPTTLRPKLFGQNLEGDLPIARRKSLQRFLEKRKERLVSTSPYYPTSA

>AtJAZ11（At3G43440）

MAEVNGDFPVPSFADGTGSVSAGLDLLVERSIHEARSTEPDASTQLTIIFGGSCRVFNGVPAQKVQEIIRIAFAGKQTKNVTGINPALNRALSFSTVADLPIARRRSLQRFLEKRRDRSTKPDGSMILPSQLTIIFGGSFSVFDGIPAEKVQEILHIAAAAKATETINLTSINPALKRAISFSNASTVACVSTADVPIARRRSLQRFFEKRRHRFVHTKPYSATTSEADKNETSPIVT

>AtJAZ12（At5G20900）

MTKVKDEPRASVEGGCGVADGDGGAAEIGGTGSVEKSINEVRSTEIQTAEPTVPPNQLTIFFGGSVTVFDGLPSEKVQEILRIAAKAMETKNSTSISPVSSPALNRAPSFSSTSNVASPAAQPFPIQPISFCRSTADLPIARRHSLQRFLEKRRDRLVNKNPYPTSDFKKTDVPTGNVSIKEEFPTA

>AtJAZ13（At3G22275）

MKGCSLDLHLSPMASTLQSCHQDSTVNDRSSTIRSKEINAFYSGRLSEYDLVEIQMRAIIEMASKDREVTALELVPVRLESPLGCSVKRSVKRFLEKRKKRSKSFTLTPNYTSSTSSSSSSLHNF
