# Supplementary material for: Genome-wide identification and expression analysis of jasmonate ZIM domain gene family in tuber mustard (Brassica juncea var. tumida)
Source: PLoS One. 2020 Jun 16;15(6):e0234738. doi: 10.1371/journal.pone.0234738 (PMC7297370; doi:10.1371/journal.pone.0234738)
Supplement: S2 File — (DOCX) [file pone.0234738.s005.docx]

**Supporting information file 1:** the peptide sequences of JAZ proteins in the genome of *Arabidopsis* and *Braassica juncea* var. *tumida*

> BjuB032431

MSSPMESSDFAATRRFSRKPSFSQTCSRLSQYLKENGSFGDLSLGMACKPEVNGISRQPTTTMSLFPCEASNMEPIGQDVKPKNLFPRQPSFSSSSSSLPKEDILKMTQATSSTRSVKPEPQTAPLTIFYGGQVIVFNDFSAEKAKEVMDLASKGTANTFTGFTSNVNNNIQSVYTTNLANNQTEMRSNIAPIPNQLPHLMKITTQNPVQSSSTAMACELPIARRASLHRFLAKRKDRVTSKAPYQLNDPAKASSKPQTGDNTTSWLGLAAEM

> BjuB031487

MSSPMESSEFAATRRFSRKPSFSQTCSRLSQYLKENGSFGDLSLGMACKPEVNGISRQPTTTISLFPCNMDSMEAGQDVKPKNLFPRQPSFSSSSSSLPKEDVLKMTHTTTITRSVRPEPQTAPLTIFYGGQVIVFNDFTAEKAKEVMDLASKGTANTFTGFTSNVNNNNIIQSVYTSNLSKNQTEIRSNIAPIPNQLSHPMKTITQEPIQSNSTAMACELPIARRASLHRFLAKRKDRVTSKAPYQLSDPSKASSKPQTGDNNTSWLGLAAEM

> BjuA025820

MSSSMECSATRRSSSGKPSFSLTCSRLSQYLKENGSFGDLSLGMSCKPETNGMSRKPTTTMSLFPCEASNVGSMAAAQDVKPKNLFPRQPSFSSSSSSIPKEDVPKIAQTTTTRSLKPEPQTAPLTIFYGGQVIVFNDFSAEKAKEVMNLASKGTANTFTGFTSTLNNNIAPTPNQVPHLMKTASQDPKQTSSAAMACELPIARRASLHRFLAKRKDRVTSKAPYQLSDPAKAFSKPQTGNSTTSWLGLAADM

> BjuB043409

MMSSSMECSDSGATTRRFSRKPSFSLTCSRLSQYLKENGSFGDLSLGMSCKPEVNGNSRQPTTTMSLFPCEAPSMATGQDVKPENLFSRQPSFYSSSSSLHKEDVLKTITTTISVKPEPQTAPLTIFYNGEVIVFDDFSAEKAKEVMNLASKGTANTFTGFTSTVNFAKNQTEVRSNIPHLVKTAAQEPISTAMACELPIARRASLHRFLAKRKDRVTSKAPYQLSDPARASSKPQTGDNATSWLGLAAQI

> BjuA030800

MMSSSMECSDSAATRRFSRKPSFSLTCSRLSQYLKENGSFGDLSLGMSCKPEVNGISRQPTTTMSLFPCEAAQDVKPKNLFPRQPSFSSSSSSLPKKEEVLKMTQTTTTRSVRPEPQTAPLTIFYNGEVIVFNDFSAEKAKEVMDLASKGTANSFTGFTSTVNLPKYQTEVRTNISPTLDQVTHLMKPAAQEPILSSSAAMACELPIARRASLHRFLAKRKDRVTSKAPYQLSDPAKASSKPQTGDNNTTSWLGLAAQI

> BjuB021388

MSSSAECWEFSSRKLPEKPSFSQTCSRLSRYLKEKGSFGDLNFVMTMTCKPDVNASGIDSEAAQDVKLKNDMFPWQSSFTSFSSFGAKEEVEKIKQTKDVKPESQSAPLTIFYGGQVMVFDDFPAEKANQVIDLASKGSANGFTAELNNNKSAYPGNLAKKQKEIASIPRPVPSPAKTAAQEPIQTNTSSLASELPIARRASLHRFLEKRKDRITSKAPYQKEGSTEA

> BjuA027037

MSSSAECWEFSGPKLPEKPSFSQTCSRLSRYLKEKGSCGDLSFSMTSKPDVNASGINSKAAQDVKLQNDMFPCQSSFSSSFGVKEEVVKITETKPVKPESQSAPLTLFYSGQVMLFDDFPAEKAKQVIDLANKGSANGFTAELNNNQSAYTKNIAKNQKEIASIPRPVPSPAKKPAQEPIQTNTSSLASELPIARRASLHRFLEKRKDRITSKGPYQKEGSTEA

> BjuA029428

MSSSAECWEFSGERKRLLEKKPSFSQTCSRLSRYLKEKGSFGDLSLGMTCNGGGNLAVTRQQPTMMNLFPVEDSSDVKQKNDVIPRQSSFSSSSSSGAKEDVEKITETKSVKVESQSSAPLTIFYGGQVMVFDDFPVEKAKQVIDLAHKGSAKSFTAELNIAKNNHKEIASTTPVPVPSPVKTAAPEPIQTNKSSLACELPIARRASLHRFLEKRKDRISSKAPYQIDGSTEASSKPSTALLGSQ

> BjuB029798

MSSSAECWEFSGQRKRLLEKKPSFSQTCSRLSRYLKEKGSFGDLSLGMTCNPNVNGVFGVSRHPTMMNLFPVEDSSAGQDVKPKNDVFPRQSSFSSSSSSGGVKEEVEKIIQTKSVKPEPQSAPLTIFYGRQVMVFDDFPAEIAKQVIDLAHKGSAKSFTTELNNNQSASTQKEIVSTTPNPVLRCPVKTAAPEPIQTNKPSLACELPIARRASLHRFLEKRKDRITSKAPYQIDGSTEASSKPNTAWPGSQ

> BjuA005572

MSSSAEYREFSGRKLQKTPSFSQTCSRLSRYLKEKGSFGDLSLGMTCNPDVTGVFAVSRQPTMMNLFPCEEASPTQDVKPTHKVPRQSSFSSSSSAGAKGEVEKIIETKSVKVESQSAPLTIFYGGQVMVFDAFPAEKAKQVIDLANKGSDYAQNIAKNQKEIASTTPNPVPSLAKTAAAPELVQTNTSSLACELPIARRASLHRFLEKRKDRITSKAPYQIDGSTEASSRPDTSWLGSQ

> BjuB011370

MSSPAECRKLQKKPSFSETCSRLSRYLKEKGSFGDLSLGMTCSPDVNGVFAVSRQPTVMNLFPCEDASSPQNVKPKDDMVPRQTSFSSSSSSGAKEEVEKIIETKSVKVESQSSPLTIFYGGQVMVFDDFPAEKAKQVIDLANKGSAESSELNKNQSAYTQKLAKNHKDIASATPNPSPAKTTATQEPLQNNTSSLACELPIARRVSLHRFLEKRKDRITSKAPYQIDGSTEASSKPNTSWLGSQ

> BjuA045157

MERDFLGLGSKNSPITVKEETSESSRDSAPSRGMKWSFPNKASATSAPQFLAFRPSQENRHRNLGNYHLPHSGSFMPSSIADVYDSSNRSTPYSSVQGVRMFPSSKQHEEAISVSMSRPGLQSHYASGGTSFINNSVNSQPLVGVPIMAPPVSVLPPPGSIVGTTDIRCSSKPSGSSPAQLTIFYAGSVCVYNDISPEKAKAIMLLAGNGSPMPQAFSPPQTHQQVVHHARASVDSSAMPPSFMPTVSYLSPEAGSSSNGFGAAKAARGFRTTYLSNQTNASNINSSVAASCSANVPQTVALPQARKASIARFLEKRKERVTSLSPYCLDKKSSTDCRTPMSECISSSLSSAT

> BjuB007213

MERDFLGLGSKNSPITVKEETSESSRDSAPSRGMKWSLSNKASATTSSPQFLAFRPSQEDRHRNIGNYHLPHSGSFMPSSVADVYGSNRSTPYNSVQGVRMFPSSKPHEEAISVSMSRPGLKSHYAPGGTSFINNNVNSQPLVGVPIMAPPVSVLPPPGSIVGTTDIRCSSKPSGSSPAQLTIFYAGSVCVYNDISPEKAKAIMLLAGNGSSMPQAFSPPQTHQQVVHHARASVDSSAMPPSFMKPTVSYLSHEAGSSSNGFGAAKAARGFTTTYLNNQTNASNINSSAAVSCSANVPQTVALPQARKASLARFLEKRKERVTSVSPYCLDKKSSTDCRTPMSECISSSLSSAT

> BjuA046021

MERDFLGLGSKNSPITVKEETSESSRDSAPNRGMNWSFSKKGSAASSQFLSFRPSQDDRHRKPGNYHLPHSGSFMPSSVADVYDSNRNTPYSSVQGARMFPNSHQQQESITVSMARPGLQSHYPPGGKSFMSNGINSQPFVGVPIMAPPISVLPAPGSIVGTTDIRSSSKPLGSPAQLTVFYAGSVCVYDDISPDKAKAIMLLAGNGSSMPQAFSPPQTHQQVVHHARASVDSSAMPPSFMPTVSYLSPEAGSSTNVLGARGFASTYHNNQTNASTVKPQTVALPQARKASLARFLEKRKERVTSVSPYCLDKKSPTDCRTPISECISSSFSSAT

> BjuB025543

MERDFLGLGSKNSPITVKEETSESSRDSAPNRGMNWSFSKKGSAASSQFLSFRPSQDDRHRKPGNYHLPHTGSFMPSSVADVYDSNRNTPYSSVQGARMFPNPNQQQESITVSMARPGLQSHYAPGGKSFMSNGVSSQPFVGVPIMAPPISVLPAPGSIVGTTDIRSSSKPLGSPAQLTIFYAGSVCVYDDISPDKAKAIMLLAGNGSSMPQAFSPPQTHQQVVHHARASVDSSAMPPSFMPTASYLSHEGGSSTYGLGAVKATTGFTSTYHSNQTIAPTVKPQTVALPQARKASLARFLEKRKERVTSVSPYCLDKKSSTDCRTPISECISSSFSSAT

> BjuB043343

MSRNEDGKAQPQEKFNFTRRCSLLSRYLKEKGSFGNIDLGLVRKPESDLKLPGNSDQQEKQNVMHKANSELKALNVLGEPSSSSGGKAKATNLSEPSDPGSSQLTIFFGGKVLVYNEFPVDKAKEIIQVAKQAKPVTEVTVQNNNKSNMVLPDLNEPTDSADVNQQQQQQNQLVERIARRASLHRFFAKRKDRAVARAPYQVNQSTGRHHYPPKPNNVPGQQLEQGQSSQRPAQSKPECDKDMLMEVNEEGQCSKDLELRL

> BjuA022138

MSRNENAKAQPLEKSNFTRRCSLLSRYLKEKGSLGNINLGLIRKPNPPGKQHKADSETKTLDVFQRVLKGEPSPGKANEDSNLSSQLTIFFGGHVLVYNEFPTDKAKEILEVAKQAKPVTDINIKTQINVENNDNKSNMVLPDLNEPTNSVDIINQQNQVVERIARRASLHRFFAKRKDRAVARAPYQVNQNVGQHHYPPKPETAHGRSLKSGQSSKAPEEDVAQTMSQPKPEGDKYMSIETEEEGQCSKDLQLRL

> BjuB029203

MSRNVSNENAKAQPLEKSNFTRRCSLLSRYLKEKGSFGNINLGLVRKPNSDRVLSGNSDPPVKQHKADSETKALFRMISKGETSPSSRGKAKEDTNLRFWFNFSKSSRPGNSQLTIFFGGQVLVYNEFPTDKAKEIMKVAKQAKPVIDINIKTQINVENNKSNMVLPDLNEPTNSVDIINQQNHVVERIARRASLHRFFAKRKDRAVASAPYQVILPSQRLPTVNHLNQGSRQNDRRMLLLKPCPTQNQKATNICL

> BjuA030507

MSRNEDGEAPPPEKSNFTRRCSLLSRYLKEKGSFGNIDLGLVRKPGPDLGLPGNSDQQEKQNVMHKANSELKALNVLGEPSSSFGGKAKATNLSEPSEPISSQLTIFFGGKVLVYNEFPSDKAKEIIQVAKEAKSVTDINIQTQINVQKDHNKSNIVLPDLNEPTDTADVNQQQQQQNQLVERIARRASLHRFFAKRKDRAVARAPYQVNQNGGGHHYPPKPETVPGQQLEQGQSSQPQRPAQPKPECDKDMLMEVKEEGQCSKDLELRL

> BjuA001107

MRSTPIPLVLSHSTSKTRTNKKKKIAIKTRKDSGEFLLSDTYKMSRKENAKALGPPLEKSSFTRRCSLLSRYLKEKGSFGNINLDLIRKPDSDLGLPGYSCPPGSFGEQSLRMYSGDSKAEPSPHRSKARYNSGLYYLLSFDIKPASDSGSSQLTIFFGGQVLVYNEFPADKAKEIMEVAKKAKPVTEVNIQTQINVENNNTNNIQTQINVENNNNNKSNMVLPDLNEPTDSMDINPQQQQENQVVERIARRASLHRFFAKRKDR

> BjuB030035

MLGYPFTRSVQQNAIKKADISETRPFKLTQKQLSVGETSTSRGKAIDVDLSEPTNVPEPKISQLTIFFGGKVIVYNEFPEDKAKEIIEAAKEAHPVVVDSNNTEDHRNINNKRNVVIPDLNEPVSSGNKDDHQTKEQHQVVERIARRASLHRFFAKRKDRAVARAPYKVNQNGGHLPPKPQMVRPSAESGQPSRQPATPSKPKSRDDMSMAVEEEEGRCSKDLELKL

> BjuB010656

MSAGKAPEKSTFSRRCSLLSRYLKEKGSFGNIDIGFAHKLDLDLAGKSDLRGQQNEIKKAYISDTRPFDLIQKVSVGEASTSSGGKPRYVDLSEPARVVPETGNSELTIIFREKIMVYDQFPEDKAKEIIEAARKAHRVAVDSKNSQNLDMNMSNKRNVVIPDLNEPTSSETNDDDHQTGQQHQVVERIARRASLHRFFAKRKDRAVARAPYQVNQNCGHLPPKPPMVGPSAEPGQPSRQPLSPPKPKIHNDMSMEVDGEEGRCSKDLDLKL

> BjuA007483

MSNGKAPEKSSFSRRCSLFSRYLKEKGNLGNIDIGLSRNLDLELVRKSDLSGQQNEIKKADISETRPFDLSQKVSVGEASTSSGGKPRFVDLSEPASLVVPEPGNSQLTIFFRGKVMVYDEFPEDKAKEIMAAAREAHHVAVDSKNTQNLDMNMSNKTNVVIPDLNEPTSSGTNNDDHQTGQQHQVVERIARRASLHRFFAKRKDRAVARAPYQVNQSGGHLPPKPQKVGPSVESGQPSRQPETTSKPKRHNDASMEVDGEEGRCSKDLELKL

> BjuB026559

MEMQSNCDLELRLVSPPYDSSSSKTPQPKKESQILTIFYNGHICVSSDLTHPQAKAILSLASKDMEEKPLSLKSSDGPDPPIIPNNLTQVHQQKASMKRSLRSFLQKRNVRIQASCPYHHSR

> BjuB032915

MEKNCDLELRLFPTSSYDTDSDTSVVESRSSGNSLPKEEESQRITIFYNGKMCVSSNVTHLQAKSIISIASREMEERSSSNGSDPQNRLTRLHHQQLPNPKASMKRSLQSFLQKRRIRIQATSPYHQQSRR

> BjuB029529

MEKNCDLELRLFPTSSCITDSENSVVESRSSGNSLPKEEETQRLTIFYNGKMCVCSDVTHHQAKSIISMASTEIEERSSSYGSDPRNRSTRLNHDHRQLPNPKASMKRSLQSFLQKRQIRLQATSPYHQRR

> BjuA034780

MEKNCDLELRLFPTSSCITDSDNSVVESRSSGNSLPKEEETQRLTIFYNGKMCVYSNVTHHQAKSIISMARREMEEKLSSNVSDPRNRSTQLNNYHQQLPNPKASMKRSLQSFLQKRQIRLQAASPYHQHSRQ

> BjuA007387

MERDFLGLSDKKYLSNVKREANDDRVGERVLSKKAAIQWGKAKLLPNSSFMPDFQAGSYQRGPVSAASNLRRSQFSGGAFQNANPLLLGGSVPLTNHSSFRPAFNLSADARVASSGSLPQLTIFYGGTVSVFNNISPDKAQAIMLCARNGLQGETGESSLKKPVQETERVYGKQVHNAAAAASSSSATYADSFSRCRDIPVGATNAMSMIESFNAAGPGNMIPSVPQARKASLARFLEKRKERLMSALPYKKMLLDLSTGESSGMNYSSASHT

> BjuA027135

MERDFLGFSDKQYLNNVDDDRVGERGSSTKAARQWGPESAASIHRRSQHSGAFQNANPQLTIFYAGTVCVFNDISPDKAQAIMLCAGNGLKIDNGESRLKKPLIETERVYGKQFHNAATAAASSSSATYCDNFSRCGDRPVGATNAMSKIESFNVDPGYMMPSVPQARKASLARFLEKRRERLMNAMPYKKMLLDLLTRESYGMNYSSASHT

> BjuB030369

MERDFLGLSDKQYLSNVKREVDGERVGEGGLSKKAAKQWGKAKLLPSSSYMAAAADFQMSRSHATPGSYQWCPVYAASTHRRSPFGGGAFQNVNPLLLGGSVPLTNHSALRPAFNSSTDPRVASSGSSPQLTIFYAGTVTVFNDISPDKARAIMLCAGNRSKVETGESSLKKPLRETERVYGKQIHKATPAASSSSATYADSFSRCKDRHVGATNAMSMMESFNAGPSNMIPSVPQARKASLARFLEKRKERIMSAMPYKKMLLDLSTAESSAASHT

> BjuA027422

MERDFLGLSDKQYLNNVKPDVDDDRVGERGLSKKIAKQWGKAKLLPNSSFMPAAVDLQWCPVSAASIHRRSQFGGGAFQNANQLLLGGSVPLTNHSALRPAFNSSRDPRVASSGSSPQLTIFYAGTVTVFNDISPEKARAIMLCAGNGLQGENGESSLKKPLRETERVFYGKQIHKATAAASSSSATNADSFSRCKDKHVGATNAMTMTIESFNAGPSNMIPSVPQARKASLARFLEKRKERIMSAMPYKKMLLDLSTGESSAASHT

> BjuB035964

MSKVTTELDFFGLEKKQTNNAPNPKFKKFLDRRRSFREIQGAISKMDPEIIKSLLASGANPSDTFTRSPSVPSTPKEDHPHIPISPVHAPLTRPGMVPVSGTVPMTIFYNGTVSVFQVSPNKAEDIMKVAMETSPKRDKSMEKDLPVIPQTTLRTKLFGQNLDGDLPIARRKSLQSFLEKRKERVVSTSPYFPTSA

> BjuA041687

MSRVTTELDFFGLEKKQTNNVPKPKFKKFLDRRRSFRDIQGAISKIHPEIIKSLLASGANHADSSTISPSVPSTPKADHPQIPISPVQAPLTMQTEHSSGTVPMTIFYNGTVSVYQVSPNQADDILKVVMETAPKKDKSIVKDHLVIPPTTLRTKLFGKNLEGDLPIQRTRSLQRFLEKRKERLVSISPYFPTSG

> BjuB014771

MSKVTTELDFFGLEKKQTNNVPKPKFEKFLDRRRSFRDIQGAISKINPEIIKSLLASGANHPDSSTISLSVPSTPKADHPQIPISPIQAPLNMPTELVSGTVPMTIFYNGTVSVYQVSPNKADDIMKVVKETAPKKDKSIAKDHSVIPQTTLRTKLFGQNLEGDLPIQRTKSLQRFLEKRKERLVSTFPYFPTSA

> BjuA022588

MVKVEDEPRAPVEGGCGNIVGRGVVDGDGGEENHVVEIAGDATVNGIIAGSVAGEGEFSAEKVAQEARSTDAPSSDVPDPSTILPNQLTIFFGGKVCVFDGIPAEKIQEIIRIAAAATAKSIETKNSTSVKPVLSPALNRAPSFSSTSTGASPAAPSLPVNPIPFCRSAADLPIARRHSLQRFLEKRRDRLVNKNPYPASDMKKTDVPTDIASIKEESPIA

> BjuO008948

MTMVKVEEEPRAPVEGGCGSDGGEEHRRVEIGGNVTVHGSIAGSVAGKGAEKQIHEARSMEVPSSETDATTIRPNQLTIFFGGKVRVFNGIPADKIQEIIRIAAAAAKSVETKNSTNTSPVASPALNRAPSLSSTSNAAASPPAQSFPIHPISFCRSAADLPIARRHSLQRFLEKRRDRLVSKNPYPASDKKTDVPRDNASIKEEEYPTA

> BjuA047148

MTMVKVEEEPRAPVEGGCGVGGEEIGGNGTVHGSIAGSVAGEGAEKETHEARSLEVPSSEPDASTTRPNRLTIFFGGKVRVFDGIPADKIQEIIRIAAAAAKSIETKNSANTSPVASPALNRAPSLSSTSNAAASPAAQSFPIHPISFCRSAADLPIARRHSLQRFLEKRRDRLVSKNPYPASDKKTDVPRDDASIKEEYPTA

> BjuA001950

MENRSLDLCLSSVTSSLQSCRRDSKVSQSLATRTKEINAFYSGRVREYDLVEIQIRAVIEMASKERDITALELAPVRLKSPLVFSVKRSVERFLEKRKKRSKYVTTPYGYTCSSTSSSSSRHS
